# A Pilot Study of Biomedical Text Comprehension using an Attention-Based Deep Neural Reader: Design and Experimental Analysis

**DOI:** 10.2196/medinform.8751

**Published:** 2018-01-05

**Authors:** Seongsoon Kim, Donghyeon Park, Yonghwa Choi, Kyubum Lee, Byounggun Kim, Minji Jeon, Jihye Kim, Aik Choon Tan, Jaewoo Kang

**Affiliations:** ^1^ Department of Computer Science and Engineering College of Informatics Korea University Seoul Republic Of Korea; ^2^ Interdisciplinary Graduate Program in Bioinformatics Korea University Seoul Republic Of Korea; ^3^ Division of Medical Oncology Department of Medicine Translational Bioinformatics and Cancer Systems Biology Laboratory, University of Colorado Anschutz Medical Campus Aurora, CO United States

**Keywords:** machine comprehension, biomedical text comprehension, deep learning, machine comprehension dataset

## Abstract

**Background:**

With the development of artificial intelligence (AI) technology centered on deep-learning, the computer has evolved to a point where it can read a given text and answer a question based on the context of the text. Such a specific task is known as the task of machine comprehension. Existing machine comprehension tasks mostly use datasets of general texts, such as news articles or elementary school-level storybooks. However, no attempt has been made to determine whether an up-to-date deep learning-based machine comprehension model can also process scientific literature containing expert-level knowledge, especially in the biomedical domain.

**Objective:**

This study aims to investigate whether a machine comprehension model can process biomedical articles as well as general texts. Since there is no dataset for the biomedical literature comprehension task, our work includes generating a large-scale question answering dataset using PubMed and manually evaluating the generated dataset.

**Methods:**

We present an attention-based deep neural model tailored to the biomedical domain. To further enhance the performance of our model, we used a pretrained word vector and biomedical entity type embedding. We also developed an ensemble method of combining the results of several independent models to reduce the variance of the answers from the models.

**Results:**

The experimental results showed that our proposed deep neural network model outperformed the baseline model by more than 7% on the new dataset. We also evaluated human performance on the new dataset. The human evaluation result showed that our deep neural model outperformed humans in comprehension by 22% on average.

**Conclusions:**

In this work, we introduced a new task of machine comprehension in the biomedical domain using a deep neural model. Since there was no large-scale dataset for training deep neural models in the biomedical domain, we created the new cloze-style datasets Biomedical Knowledge Comprehension Title (BMKC_T) and Biomedical Knowledge Comprehension Last Sentence (BMKC_LS) (together referred to as BioMedical Knowledge Comprehension) using the PubMed corpus. The experimental results showed that the performance of our model is much higher than that of humans. We observed that our model performed consistently better regardless of the degree of difficulty of a text, whereas humans have difficulty when performing biomedical literature comprehension tasks that require expert level knowledge.

## Introduction

The rate of discovering and accumulating new biomedical knowledge continues to increase rapidly due to technological advances. Most of the new findings are published in the form of biomedical literature. The rate of increase in PubMed volume reflects such a growth trend. On average, more than 3000 papers are newly added to PubMed every day. As the number of publications of biomedical research papers rapidly increases, it becomes more difficult for biomedical knowledge workers to collect and assemble information from the fast-growing literature to compose answers to biomedical questions [1]. To address this issue, automatic information-seeking and processing approaches such as information retrieval, biomedical text mining [2-5], and biomedical question answering (QA) systems [6-11] have been rigorously studied in recent years.

Recently, advances in artificial intelligence (AI) based on deep learning technology not only improved the performance of existing text mining models, but also reached a level where machines can read and comprehend texts so that they can respond to given questions. In the AI community, researchers have actively conducted studies to measure a machine’s ability to understand text in reading comprehension tasks [12-17]. Machine comprehension tasks can be defined as testing the ability of a machine to answer a question based on context. Recent studies show that deep neural network-based models hold promise for performing reading comprehension tasks, and currently outperform all alternative models [12-14]. Several AI research groups, including Google, Facebook, and IBM Watson, developed new text comprehension models [12-15].

Deep learning-based approaches require a sufficient amount of data to train a model. Therefore, in addition to model architecture, methods that automatically generate a considerable amount of data (which can be used for training neural models) have been actively studied. One study used cloze-style [18] QA pairs that were employed to assess the learning ability of elementary school students. Several large cloze-style context-question-answer datasets have also been introduced. These datasets contain only general information from sources such as news articles (Cable News Network [CNN]/Daily Mail) and children’s books, and not professional knowledge.

With a well-developed machine comprehension model, one can quickly and efficiently find the correct answer to a question using the given context. However, while machine comprehension is actively studied in the AI research field, recent machine comprehension technologies have not been applied to the biomedical domain, which requires information processing the most. Currently, there are no datasets for biomedical text comprehension tasks, and thus a computer’s ability to comprehend biomedical domain knowledge has not yet been verified.

In this article, we propose a machine comprehension task on biomedical literature. We also provide a new and large cloze-style dataset called BioMedical Knowledge Comprehension (BMKC) which can be employed to train deep neural network models. Our goal was to test whether a machine can correctly comprehend scientific papers such as those in our dataset, since it has already been proven in previous research that it can comprehend general text such as storybooks. We demonstrate that our state-of-the-art deep learning model enhanced with biomedical domain-specific features can comprehend biomedical literature. Through a performance comparison with humans, we observed that the comprehension performance of humans varies depending on the degree of difficulty of a text, while machines perform consistently well.

This research offers three contributions to the field. First, to the best of our knowledge, this work is the first to propose a deep learning-based machine comprehension task in the biomedical domain. Second, we used the PubMed corpus to generate considerably large datasets for training deep neural machine comprehension models. The automatically generated datasets open huge opportunities for data-hungry techniques such as deep-learning and future QA systems. We made the datasets publicly available [19]. Third, we present methods that can improve the performance of existing machine comprehension models using pretrained Word2Vec and entity type embedding features. We employed an ensemble approach of combining multiple single models to produce improved answer prediction results. The experimental results showed that our proposed methods can help our model, based on the original text comprehension model developed for general text, to achieve state-of-the-art performance in biomedical literature.

## Methods

In this section, we first explain the process of automatically creating a large-scale biomedical text dataset for machine comprehension tasks. We then describe the Attention Sum Reader (ASR) [15], a state-of-the-art deep neural model that is used for machine comprehension tasks. We propose two additional techniques utilizing pretrained word vector and entity type embeddings, both of which we used to build our text comprehension model tailored to the biomedical domain. To improve the prediction accuracy, we also applied ensemble learning in which the final answer prediction was obtained by integrating the output of several independent homogenous models.

### Cloze-Style Biomedical Machine Comprehension Task Overview

A cloze-style question is formed by removing a phrase from a sentence; cloze-style questions are *fill-in-the-blank* type questions. The cloze-style dataset is in the form of context-question-answer triplets. From the perspective of machine learning, this task is easy to evaluate. The cloze-style text comprehension task can be defined as tuples of the form (*d*, *q*, *a*, *A*), where *d* is a document, *q* is a query, and *a* is the answer to query *q*, which comes from a set of candidate answers *A*. More specifically, given a document-query pair (d, q), we aim to find *a* ϵ *A* which answers *q*.

#### Cloze-Style Biomedical Machine Comprehension Dataset

Our BMKC datasets are in cloze-style form (context-question-answer) like other existing datasets. The main difference is that BMKC consists of scientific articles in the biomedical domain, which require expert knowledge for comprehension, while other existing datasets contain nonscientific, general texts such as news articles and children’s storybooks [12,13,16].

We explain in detail the method for generating the dataset as follows. First, we needed a document for the context. We chose the abstract of a paper as the context *d* in our BMKC datasets. Unlike the CNN news dataset in which summaries are given, abstracts of research articles do not have such summaries. Hence, we took a different approach to automatically generating questions.

The question *q* is generated in two different ways. A question in Biomedical Knowledge Comprehension Title (BMKC_T) is constructed from the title of an academic paper because the title can be considered as a short summary of the abstract of the paper. Biomedical Knowledge Comprehension Last Sentence (BMKC_LS) uses the last sentence in the abstract of a paper as a question, inspired by Hill et al’s work [13]. In short, the BMKC datasets (Table 1) can be defined as tuples of the form (*d*, *q*, *a*, *A*), where *d* is an abstract, *q* is a title (BMKC_T) or the last sentence in an abstract (BMKC_LS), and *a* is the answer to query *q*.

#### Data Generation Process

The process of generating the BMKC datasets consisted of the following three steps. First, we gathered biomedical research articles from PubMed. Having started in the 1960s, PubMed now provides more than 24 million references to biomedical and life science articles dating back as far as 1946. We downloaded a total of 200 MEDLINE files (medline16n0813-medline16n08131012) that contain approximately 2,200,000 biomedical papers that include titles, abstracts, keywords, published year, author information, and so on.

Of the 200 MEDLINE files, we used 196 files (medline16n0813-medline16n08131008) as our training set, two files (medline16n1009-1010) as our validation set, and the last two files as our test set (medline16n1011-1012). Table 2 shows the number of articles by published years in the 200 MEDLINE files. More than 95% (2,110,444/2,208,081) of the articles were published after 2010. Note that the publication dates of the journal papers were randomly distributed across the training set, validation set, and the test set.

The next step was extracting biomedical entities to generate candidate answers to cloze-style questions. We exploited the biomedical named entity extractor in Biomedical Entity Search Tool (BEST) [20]. To increase the coverage of biomedical entities, we added Medical Subject Headings (MeSH), a hierarchical biomedical vocabulary thesaurus, for our entity extraction process. One advantage of using MeSH is that it provides a kind of entity resolution function that groups several different biomedical entity names with the same meaning into one MeSH identification (ID). Next, we replaced all entity names with their unique entity IDs. Unlike the work of Herman et al [12], we did not randomly permute the entity ID for each context. Retaining unique entity IDs allows the model to acquire background knowledge during the training process, which will improve the performance of the biomedical knowledge-specific QA task.

**Table 1:** Example of BMKC_T (Title) and BMKC_LS (Last Sentence). In the BMKC_LS dataset, the last sentence of context is excluded in training as it is a question itself.

Parameter	BMKC_T (Title)	BMKC_LS (Last Sentence)
Context (abstract of a paper)	In breast cancer, overexpression of the nuclear coactivator NCOA1 (SRC-1) is associated with disease recurrence and resistance to endocrine therapy. To examine the impact of NCOA1 overexpression on morphogenesis and carcinogenesis in the mammary gland (MG), we generated MMTV-hNCOA1 transgenic [Tg(NCOA1)] mice. (...) In a cohort of 453 human breast tumors, NCOA1 and CSF1 levels correlated positively with disease recurrence, higher tumor grade, and poor prognosis. Together, our results define an NCOA1/AP-1/CSF1 regulatory axis that promotes breast cancer metastasis, offering a novel therapeutic target for impeding this process.	
Question	___?___ directly targets M-CSF1 expression to promote breast cancer metastasis.	Together, our results define an NCOA1/ ___?___ /CSF1 regulatory axis that promotes breast cancer metastasis, offering a novel therapeutic target for impeding this process.
Answer Candidates (Biomedical Named Entities)	macrophage, carcinogenesis, morphogenesis, metastasis, disease, AP-1, tumor, lung, NCOA1, (therapy, therapeutic), recurrence, mammary gland, epithelial cells, cells, CSF1, SRC, mice, c-Fos, human, affect, (breast cancer, breast tumors), efficiency	

**Table 2:** Number of publications by years in the 200 MEDLINE files.

Year	Number of papers
1910 - 1959	12,178
1960 - 2009	85,459
2010 - 2016	2,110,444

Last, we filtered context-question pairs that did not meet the following two conditions: (1) the answer should appear at least once in both the context and the question to form a valid context-question pair, and (2) the total number of candidate answers should exceed 20 to ensure a certain level of difficulty and a fair comparison with other corpora. In the end, we obtained approximately one half million context-question pairs for both the BMKC_T and BMKC_LS datasets.

### Attention Sum Reader

The Deep Long-Short Term Memory Reader [12] was first proposed to perform a machine comprehension task on a cloze-style dataset with a deep-learning model, and subsequent studies were also conducted. Recently, attention-based models have been actively studied among various deep learning models due to their high performance on various tasks [21-24]. Since the text comprehension task involves selecting one correct word in the context, the attention mechanism achieves superior performance on the task. Specifically, the ASR model [15] achieves state-of-the-art performance on the general text datasets (CNN and Daily Mail). Hence, we performed our task of biomedical literature comprehension based on ASR architecture. The overall ASR model works as follows.

**Figure 1:**
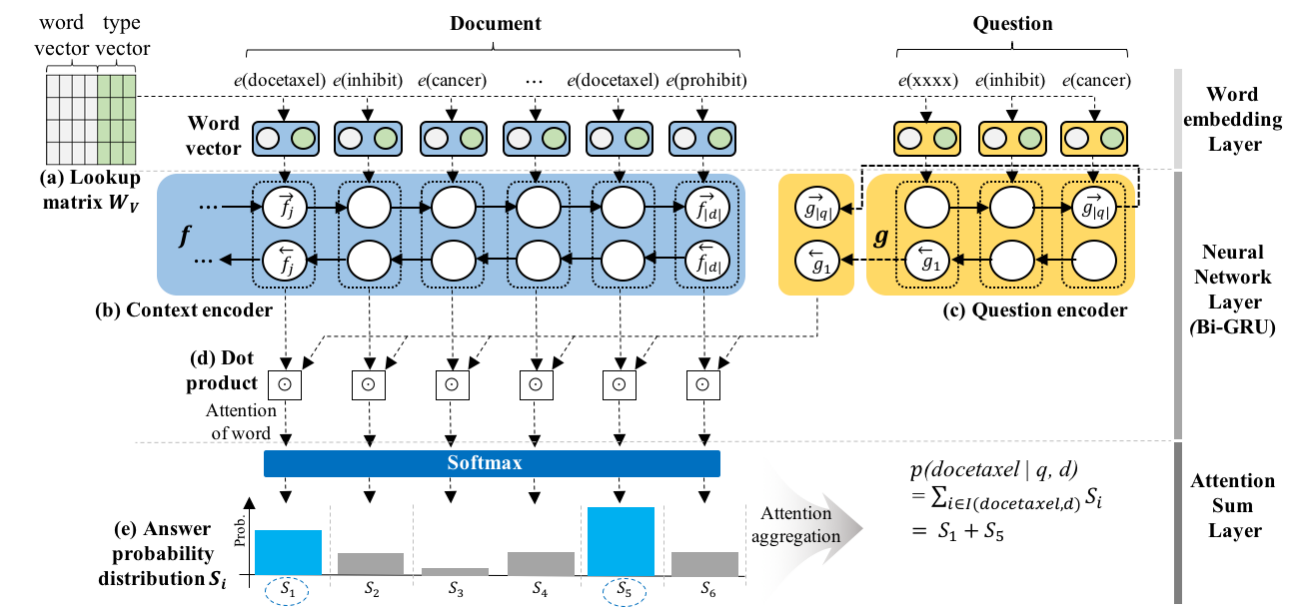
The ASR model architecture adopted from the original paper.

The ASR model uses the word embedding function e, utilizing look-up matrix W_v_, to convert words into low-dimensional vector representations whose rows are word indices from the vocabulary V (Figure 1 a).

The model has two encoders: a context encoder (Figure 1b) and a query encoder (Figure 1c). The encoders convert a context and a query into continuous vector representations. The context encoder *f* is implemented by a bidirectional Gated Recurrent Unit (GRU). Details of the answer calculation process are as follows:

The encoders receive word vectors by the word embedding function ℯ as an input. We denote the contextual embedding of the *i*-th word in *d* as *f_i_ (d)=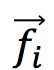
(d)||
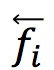
(d)* where || denotes the vector concatenation of forward and backward contextual embeddings 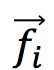
and 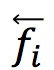. Then, a query is encoded by the query encoder *g* which is also implemented by another bidirectional GRU network such that *g(q)=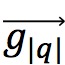
(q)||
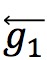
(q)*. The parameters *f*, *g*, and ℯ are jointly optimized during the training phase.

Next, word attention (answer probability) *i* is calculated by the dot product between the encoders (Figure 1d) and passed to the soft-max layer as follows:

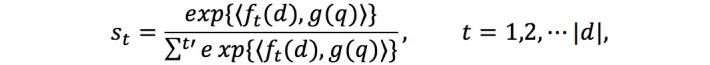



where <,> denotes the dot product between the vectors. Finally, the model calculates the scores of all possible answers based on their representations, and combines multiple mentions of the same candidate answer by adding up their answer probability (Figure 1e). The final answer token has the highest probability *P* (*a* |*d*, *q*) to answer question *q* over given document *d* such that:

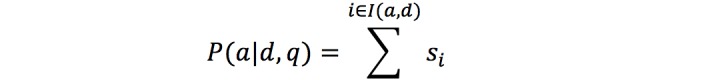



where *I (a,d)* is a set of positions of the answer token in the document. The candidate answer with the maximum probability is then selected as the final answer.

As we described, the ASR model adopts an aggregation scheme known as pointer sum attention. Hence, the performance of the attention-based model is superior to that of the general deep learning models [12,13]. Since the attention-based model is suitable for focusing on a specific target, it can achieve high performance on the cloze-style QA task of selecting a specific word to answer a question using context.

**Table 3:** The list of entity types from two information sources: BEST entity extractor [20] and MeSH tree structures.

Type Source	Entity Types
BEST	Gene, Drug, Chemical Compounds, Target, Disease, Toxin, Transcription Factor, miRNA, Pathway, Mutation
MeSH	Anatomy [A]; Organisms [B]; Diseases [C]; Chemicals and Drugs [D]; Analytical, Diagnostic and Therapeutic Techniques, and Equipment [E]; Psychiatry and Psychology [F]; Phenomena and Processes [G]; Disciplines and Occupations [H]; Anthropology, Education, Sociology, and Social Phenomena [I]; Technology, Industry, and Agriculture [J]; Humanities [K]; Information Science [L]; Named Groups [M]; Health Care [N]; Publication Characteristics [V]; Geographicals [Z]

### Improving Model Performance Using Pretrained Biomedical Word Embedding

Representing words as low-dimensional vectors is a key element of deep learning models used in natural language processing (NLP) tasks. As described in the previous section, the neural model selects the correct answer using the inner product between the vectors of the context and the query representation. Therefore, if the vector of the word that makes up the context and the query are well represented in the vector space, the probability that the chosen answer is correct will be higher.

It is known that word embeddings trained on an adequately large corpus capture latent semantic meanings and improve performance on nearly all NLP tasks. The openly available biomedical literature resources (eg, PubMed and PubMed Central Open Access) contain over 5.5 billion words in abstracts and full texts [25]. Using word embedding vectors trained on such a large amount of text can improve the performance of the model in our task. This is true because a vector representation learned on a large corpus captures more precise semantics of words. We therefore aimed to improve the performance of the original ASR model developed for general text (news) using a pretrained word vector instead of a randomly initialized word embedding. We downloaded the pretrained word vector from Pyysalo et al [26]. The details about bio-word vectors are as follows. The source data for training bio-word-vectors were derived from PubMed and all of the full-text documents obtained from the PubMed Central Open Access subset. The word vectors were generated by the Skip-Gram model with a window size of 5, hierarchical soft-max training, and a frequent word subsampling threshold of 0.001. We used 200-dimensional word vectors, as done in many previous NLP tasks. We compared the performance of each initialization of the lookup table in the *Experimental* section.

### Improving Model Performance Using Entity Type Embedding

Adding entity type information can be helpful for understanding contexts. For example, when expressions such as “@entity1 expression” or “@entity2 expression” appear in the context and the model knows @entity1 and @entity2 are *Gene* type entities, the model can learn that the context is about gene expression. Also, when other expressions such as “@entity3 0.3%” or “@entity4 100mg” appear and information that @entity3 and @entity4 are *Drug* type entities is given, the model can learn that the context is about drug concentration.

To leverage type information of biomedical entities, we used entity types identified by the BEST entity extraction tool [20]. To improve recall, we additionally extracted MeSH terms and utilized the MeSH term hierarchy as each term’s entity type label. More specifically, the MeSH tree has a hierarchical structure similar to that of concept ontology. We used parent nodes in the MeSH tree as representative entity types. Finally, we selected 10 entity types from BEST and 16 types from MeSH (Table 3).

Next, we merged some entity types that share similar semantics. For example, *Gene*, *Target*, and *Transcription Factor* types can be merged into the type *Gene*. Similarly, *Drug*, *Toxin*, *Chemical Compounds,* and *Chemicals and* *Drugs* types are merged into the representative type *Chemicals and* *Drugs* [D]. We assigned *Unknown* if words did not have a specific type. We finally constructed 20-dimensional randomly initialized type embedding vectors and concatenated them to the original word vector (Figure 1a).

### Improving Model Performance Using an Ensemble Model

A neural network ensemble approach combines the prediction results of individual models. This ensemble approach can lead to performance improvement based on its generalization capabilities [27]. Two considerations for a neural network ensemble approach are individual network generation and integrated output [28]. We adopted the ensemble averaging method in this study. An ensemble averaging consists of a set of independently trained neural network models which share the same training data, and whose individual outputs are linearly combined by averaging the results of the individual models to produce an overall prediction. Since the weights of each neural network model are randomly initialized, we can create an independent network with the same network structure. Although the resulting ensemble model has the same bias as the individual models, its variance is reduced, and thus it can achieve better prediction accuracy than a single model.

## Results

### Biomedical Knowledge Comprehension Dataset

Our BMKC datasets are the first large-scale datasets developed for biomedical machine comprehension tasks. We made our dataset publicly available for future research use [19]. Table 4 shows the statistical summaries of our dataset in comparison with four existing machine comprehension datasets.

**Table 4:** Statistics of BMKC datasets and other existing datasets. Note that the number of queries is equal to the number of documents since one query is generated per document.

Dataset		Number of Queries	Maximum number of options	Average number of options	Average number of tokens	Vocabulary Size (all)
**BMKC_T**						
	Train	463,981	93	25.6	291	876,621
	Validation	5278	66	25.4	291	
	Test	3868	74	25.7	289	
**BMKC_LS**						
	Train	362,439	90	25.3	270	714,751
	Validation	4136	57	25.1	269	
	Test	3205	74	25.4	271	
**CNN [12]**						
	Train	380,298	527	26.4	762	118,497
	Validation	3924	187	26.5	763	
	Test	3198	396	24.5	716	
**Daily Mail [12]**						
	Train	879,450	371	26.5	813	208,045
	Validation	64,835	232	25.5	774	
	Test	53,182	245	26.0	780	
**CBT_NE**^a^ **[13]**						
	Train	120,769	10	10	470	53,185
	Validation	2000	10	10	448	
	Test	2500	10	10	461	
**CBT_Noun**^b^ **[13]**						
	Train	180,719	10	10	433	53,063
	Validation	2000	10	10	412	
	Test	2500	10	10	424	

^a^CBT_NE is a dataset that uses the Children's Book Test Named Entity that appears in a context as a candidate answer

^b^CBT_Noun is a dataset that uses the Children's Book Test Noun phrase that appears in a context as a candidate answer

The CNN and Daily Mail datasets contain story-question pairs from CNN and Daily Mail news stories, respectively. The Children’s Book Test (CBT) dataset contains stories from children's books. A context consists of 20 consecutive sentences from children’s books and a question is made by removing a word from the 21st consecutive sentence. The detailed comparison of the datasets is given below. The dataset comparison is based on the training set that occupies the largest portion of each dataset.

#### Dataset Size

The size of the BMKC datasets (BMKC_T: 463,981, BMKC_LS: 362,439) is larger than that of all other datasets (CNN: 380,298, Children's Book Test Noun Phrase [CBT_Noun]: 180,719, Children's Book Test Named Entity [CBT_NE]: 120,769) except that of the Daily Mail dataset (879,450). Although the current BMKC dataset is large enough to train a reasonably complex deep neural reader, the size of the training set can easily be increased by adding articles from MEDLINE.

#### Query Length

As with the length of each query (a single context-question pair), the average number of tokens of our BMKC dataset (BMKC_T: 291, BMKC_LS: 270) is smaller than that of other datasets (CNN: 762, Daily Mail: 813, CBT_Noun: 470, CBT_NE: 433). The length of abstracts of academic papers is usually limited, while news articles can include lengthy context and have no length limit.

#### Number of Candidate Answers

The average number of options (which is the number of candidate answers to a question) of the BMKC dataset is comparable to that of the CNN and Daily Mail datasets, and larger than that of the CBT_Noun and CBT_NE datasets.

**Table 5:** Accuracies of the original ASR model and feature-enhanced models (ASR+BE, ASR+TE, ASR+BE+TE) on the BMKC_T and BMKC_LS datasets. The results of both the single and ensemble models are reported. The best scores are highlighted in italics.

Model		BMKC_T			BMKC_LS		
		Validation (%)	Test (%)		Validation (%)	Test (%)
**Single**						
	ASR [15]	79.8	77.8		73.4	70.5
	ASR+BE	81.0	*78.6*		74.6	71.4
	ASR+TE	80.9	78.5		74.3	70.1
	ASR+BE+TE	*81.4*	78.3		*74.8*	*72.0*
**Ensemble**						
	ASR	83.7	81.4		77.6	75.8
	ASR+BE	85.2	83.3		*80.1*	*77.7*
	ASR+TE	85.2	*83.9*		79.5	76.6
	ASR+BE+TE	*85.5*	83.6		*80.1*	77.3

#### Unique Vocabulary Size

The size of a unique vocabulary of the BMKC dataset exceeds that of all other datasets because academic articles contain considerably more domain-specific terms than general texts.

### Deep Neural Model Performance

#### Performance Enhancement With Biomedical Domain Specific Features

The ASR model used stochastic gradient descent with the Adaptive Moment Estimation update rule and learning rates of 0.001 and 0.0005. The model used GRU for its Recurrent Neural Network. The initial weights in the word embedding matrix were randomly and uniformly drawn from the interval (-0.25, 0.25). We used a batch size of 32.

The performance of text comprehension models on the BMKC_T and BMKC_LS datasets is summarized in Table 5. We have created four single models and four ensemble models. The ASR model represents the basic implementation of the ASR model originally developed for general text comprehension tasks [15]. The ASR model uses all randomly initialized word vectors. The ASR+Bio-word Embedding (ASR+BE) model represents an ASR model that is initialized with word vectors pretrained on PubMed, whereas the ASR+Type Embedding (ASR+TE) model represents an ASR with type information embedding. The ASR+BE+TE model denotes ASR with bio-word vector embedding and type embedding.

##### Single Model

We report the performance of the single models on the validation and test sets. While the original ASR model achieved accuracies of 79.8% and 73.4% on the BMKC_T and BMKC_LS validation set (respectively), the ASR+BE single model featuring pretrained word embedding achieved accuracies of 81.0% and 74.6% on the BMKC_T and BMKC_LS datasets (respectively), and the ASR+TE single model with entity type information obtained accuracies of 80.9% and 74.3% on the BMKC_T and on BMKC_LS datasets (respectively). The single model with all features (ASR+BE+TE) achieved the highest validation accuracies of 81.4% and 74.8% on the BMKC_T and BMKC_LS datasets, respectively. The test set accuracy also increased when we used pretrained word vectors and type embedding. The ASR+BE single model achieved the best accuracy of 78.6% on the BMKC_T test set whereas the ASR+BE+TE single model achieved 72.0% on the BMKC_LS test set.

##### Ensemble Model

We also report the performance results of our ensemble models. For the ensemble method, we used the ensemble of eight models. Among all of the learned models, we selected the model that achieved an accuracy of at least 70% on the validation set as the ensemble member. Fusing multiple models significantly increased the validation and test accuracy on both the BMKC_T and BMKC_LS datasets. As in the case of the single models, the ensemble models trained with the biomedical-enhanced features ASR+BE+TE achieved the highest accuracies on both the BMKC_T and BMCK_LS validation sets. The ASR+BE+TE ensemble model performed 5.0% and 6.6% better than the ASR+BE+TE single models on the BMKC_T and BMKC_LS validation sets, respectively (from 81.4% to 85.5% on BKMC_T and from 74.8% to 80.1% on BKMC_LS with the ASR+BE+TE setting). When using the ASR+BE+TE ensemble model, performance on the test set improved considerably. The ASR+TE ensemble model achieved the best performance of 83.9% (6.9% improved from the ASR+TE single model) on the BMKC_T test set, and the ASR+BE ensemble model achieved the best performance of 77.7% (8.8% improved) on the BMKC_LS test set.

##### Improvements From the Original ASR Model

We augmented the original ASR model [15] with bio-word embedding, entity type embedding, and an ensemble model, each of which improved the performance of the original model. The ASR+BE+TE ensemble model outperformed the original ASR model by 7.1% (from 79.8% to 85.5%) and 9.1% (from 73.4% to 80.1%) on the BMKC_T and BMKC_LS validation sets, respectively. Similarly, the ASR+BE+TE ensemble model performed 7.5% (from 77.8% to 83.6%) and 9.6% (from 70.5% to 77.3%) better than the original model on the BMKC_T and BMKC_LS test sets, respectively.

In addition, we report the top-*N* accuracy of our model’s top-*N* predicted answers in Table 6. In top-*N* accuracy, if any of the top-*N* predicted answers match the correct answer, the model’s output is considered correct. The ASR+BE+TE single model was used to compute the top-*N* accuracies. As demonstrated by the result, our model effectively puts correct answers in the top of the list of predicted answers. For example, on the BMKC_T test set, our model achieved a top-3 accuracy of 90.3%, which signifies that in over 90% of cases, users can find the correct answer in the top 3 of the outputs of the model.

#### Our Model and Human Performance Comparison

We made a test set for measuring a human’s ability to read and comprehend biomedical literature, and compared the performance of humans on the test set with that of our neural model (Table 7). For the test set, we randomly selected 25 articles each from the BMKC_T and BMKC_LS datasets. We selected articles containing the terms “human” and “cancer” that were published between 01/01/2016 and 12/31/2016.

For the human evaluees, we hired six people from three different backgrounds. The first group consisted of two undergraduate students with a background in computer science. The second group consisted of two graduate students majoring in bioinformatics. The last group consisted of two bioinformatics professionals with at least eight years of post-doctoral experience in computational oncology. To measure the comprehension ability of a machine, we used the pretrained ASR+BE+TE single model.

To evaluate the performance of the machine comprehension model, which is given a certain amount of information, we report the global ID setting in which all contexts share the global entity ID set, and the local ID setting in which the entity ID is independently assigned for each context. We provided a human evaluee with a set of tests that did not anonymize the entity ID, which is equivalent to the global ID setting for the model.

The experimental results in Table 7 show that the machine outperformed the human groups in both accuracy and time. The machine performed at a similar level to that observed in Table 6. Even in the local ID setting, in which the information about the entity is hidden from the model, the model outperformed the human evaluees. Furthermore, the human groups had some difficulty answering the given test set. The group of graduate students with biomedical background knowledge performed better than the undergraduate student group, as we expected. Interestingly, the bioinformatician group took longer to answer questions in our BMKC datasets. We assume that bioinformaticians tend to exploit their knowledge to solve the problems, whereas students with no background knowledge in the biomedical domain tend to guess. A detailed description of the test questions and the responses of our model (ASR+BE+TE) and each human evaluee is provided in the Multimedia Appendix 2.

Our model’s outperformance of humans is notable because humans have usually performed better on existing cloze-style datasets (as shown in Table 8). We present Table 8 to compare the comprehension performance of humans and the machine on the other general text domain datasets. Note that there were no human evaluation results reported for the CNN dataset when it was initially released. Hence, the CNN and CBT_NE datasets were manually evaluated by humans through the crowdsourcing platform CrowdFlower [29]. Details of the human evaluation results are provided in Multimedia Appendix 1. The results show that humans perform better than (or at least comparable to) the machine in the general text comprehension tasks.

**Table 6:** Top-*N* accuracy of the model on the BMKC test sets. The top-*N* accuracy is calculated using the ASR+BE+TE single model.

Dataset	Top-1 accuracy (%)	Top-2 accuracy (%)	Top-3 accuracy (%)	Top-5 accuracy (%)
BMKC_T-Test	78.3	86.8	90.3	93.5
BMKC_LS-Test	72.0	81.7	85.7	90.5

**Table 7:** Biomedical literature comprehension results of humans and our model on the BMKC datasets.

User		BMKC_T		BMKC_LS		Total		
		Number of problems	Accuracy (%)	Number of problems	Accuracy (%)	Number of problems	Accuracy (%)	Time (minutes)
**Human**								
	Undergraduate	14.5/25	58.0	10.5/25	42.0	25/50	50.0	77.5
	Graduate	18/25	72.0	14/25	56.0	32/50	64.0	117.5
	Expert	16.5/25	66.0	13/25	52.0	29.5/50	59.0	115.5
**Machine**								
	ASR+BE+TE_single (global ID)	23/25	92.0	19/25	76.0	42/50	84.0	0.001
	ASR+BE+TE_single (local ID)	19/25	76.0	18/25	72.0	37/50	74.0	0.001

**Table 8:** Text comprehension results of humans and the text comprehension model on the CNN and CBT datasets. The machine comprehension results are obtained from Kadlec et al [15].

Model	Dataset, accuracy (%)	
	CNN	CBT_NE
Human	69.2	81.6
Machine (ASR-single)	69.5	68.6

## Discussion

### Deep Neural Models are Less Affected by the Difficulty of the Text Than Humans

The aim of this study was to evaluate the machine comprehension model’s performance on biomedical literature datasets. In the performance evaluation on our new BMKC datasets and the existing general text datasets, our deep neural models achieved robust performance regardless of the degree of difficulty of the text, whereas humans found it difficult to solve the biomedical literature comprehension tasks that require expert knowledge. This result demonstrates that deep neural models are less affected by the difficulty of text than humans, and therefore may be used to assist human researchers when processing information in big data.

### Error Analyses

In this section, we analyzed the errors in the machine comprehension results of our machine comprehension model. The QA results of the model are shown as an attention heatmap. We discuss the two representative error cases in detail below: *causal inference error* and *concept hierarchy error*.

#### Causal Inference Error

**Figure 2:**
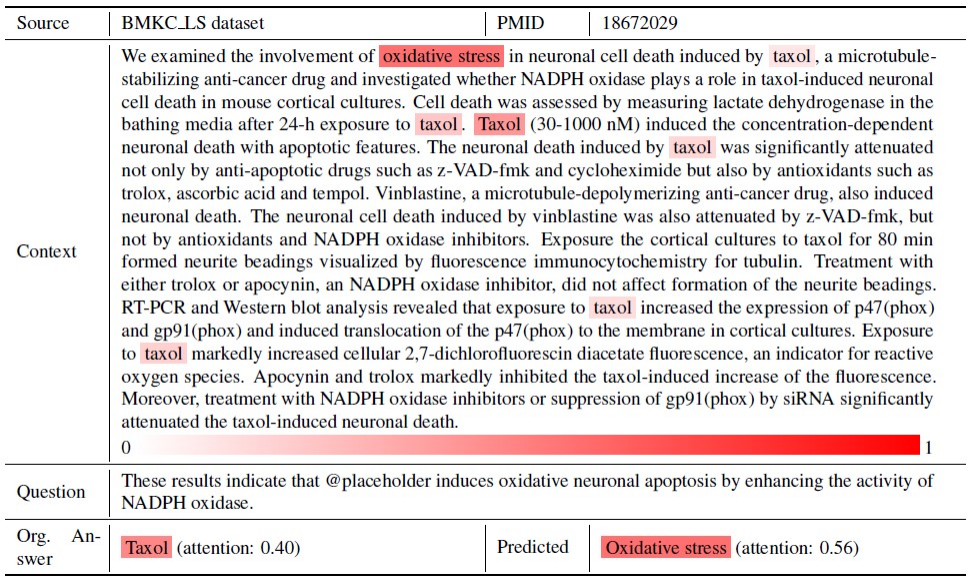
Attention heatmap from the ASR model for case 1: causal inference problem.

We observed cases in which the model could not respond accurately to questions that required step-by-step reasoning, such as a time-order relationship with the cause preceding the effect. We explain such cases using the example in Figure 2. The example document includes the relationship between Taxol, oxidative stress, and cell death. According to the context, Taxol induces oxidative stress, which leads to neuronal apoptosis. The question asked for the cause of oxidative neuronal apoptosis or cell death. As observed in the attention heatmap, the model provided oxidative stress as the cause of cell death, but it is ultimately triggered by Taxol, which is the correct answer.

#### Concept Hierarchy Error

**Figure 3:**
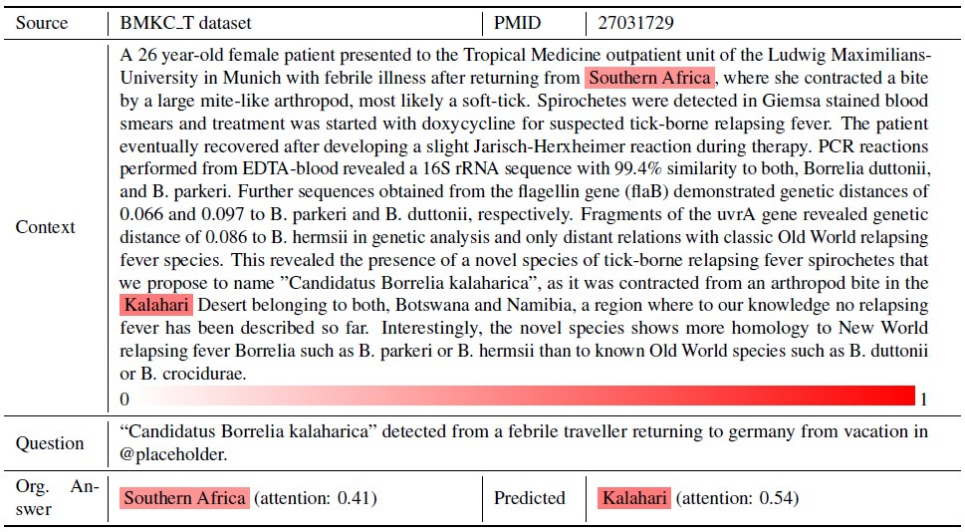
Attention heatmap from the ASR model for case 2: concept hierarchy problem.

A concept hierarchy error refers to a situation in which the model selects an option that does not match the correct answer when considering entities in an inclusive relationship. The attention heatmap in Figure 3 shows examples of concept hierarchy errors. The question asks about geo-location and the answer is “South Africa.” Interestingly, we observed that the model considers both “South Africa” and “Kalahari,” which is the name of a desert located in South Africa, as candidate answers. However, the model gave “Kalahari” more weight, which is also correct.

To summarize, the error cases discussed above can be regarded as structural limitations of the ASR model configured by the pointer-sum network method, which selects only one correct word as the final answer in the given context. The pointer-sum network structure is limited in solving questions that require an understanding of the inclusive relationship between step-wise reasoning and conceptual reasoning. Other recent deep-running models that currently perform machine comprehension tasks also do not consider such causal inference or concept hierarchy. These are fundamental limitations of the current deep learning models and should be improved with the advances of AI technology in the near future.

### Limitations of Cloze-Style Question Answering and Future Direction

The final goal of biomedical knowledge QA is to help domain experts more quickly and efficiently discover knowledge from the vast amount of information in the literature. However, the knowledge obtained through QA systems is context-insensitive and thus is not directly applicable to individual patient care scenarios. The QA systems are more appropriate to be used as decision support systems for domain experts to help them quickly process information and make more educated decisions in a shorter time.

One limitation of our current QA system is that the candidate answers are limited to biomedical entities. Although the answer probabilities are calculated for all words in the input context, the system only considers as candidate answers the biomedical entities identified by the entity extraction module used in our preprocessing step. Extracting candidate answers from the input text and providing them along with the question is a common practice in cloze-style QA systems. However, it would improve the utility of the system if the system could answer questions without prespecified answer candidates and produce any word/phrase in the text as an answer.

Another limitation that is common to all of the ASR-based deep neural models described in this paper (and other similar existing machine comprehension models) is that they assume that a single context is given when performing a machine comprehension task. During the stages of developing and evaluating machine comprehension technologies, it may be necessary to use problems that are well-defined and simple (ie, one context per question). However, such models may have limited utility in practice. If a user has a question but does not know the context or article in which the answer can be found, the user may be unable to utilize these systems. In an ideal scenario, the user should be able to query the systems without prespecifying the contexts, and the systems should be able to infer the answer by analyzing the contents of all documents in the datasets.

To address the above issues, in our future work we will expand our QA system in the following direction. First, we will modify our QA system so that it accepts a question without prespecified context and searches the entire dataset to find a subset of relevant documents. This search process can be implemented using BEST [20], which is a fast and efficient biomedical entity search tool that we developed in our previous research. Second, we will extract partial answers from each relevant document using our proposed machine comprehension model. The improved system will not require prespecified answer candidates. Finally, we will combine the partial answers from relevant contexts to form a final answer to the original query. Although searching for informative sources and expanding the proposed model to consider multiple sources would be a challenging task, we believe that this expanded system will be a useful tool for assisting biomedical scientists and practitioners by providing knowledge QA functionality in the medical domain.

### Conclusions

In this paper, we introduced a new task of machine comprehension in the biomedical domain using a deep neural model. To the best of our knowledge, our work is the first to apply the deep learning-based machine comprehension task to the biomedical domain. Since there was no large-scale dataset in the biomedical domain for training the deep neural model, we created the new cloze-style datasets BMKC_T and BMKC_LS using the PubMed corpus. To improve the comprehension performance of the existing deep neural models, we used pretrained word vectors, entity type embedding, and ensemble techniques. The experimental results show that our proposed model’s performance on the comprehension task is much higher than that of humans, including domain experts. In future work, we will expand our machine comprehension model so that it considers causal inference, concept hierarchy, and multiple documents to effectively answer complex questions.

## Appendix

Multimedia Appendix 1 Human evaluation details on the BMKC dataset.

Multimedia Appendix 2 Human evaluation of the CNN and CBT dataset using crowdsourcing.

## Abbreviations

AI: artificial intelligence
ASR: Attention Sum Reader
ASR+BE: Attention Sum Reader + Bio-word Embedding
ASR+TE: Attention Sum Reader + Type Embedding
BEST: Biomedical Entity Search Tool
BMKC: Biomedical Knowledge Comprehension
BMKC_T: Biomedical Knowledge Comprehension Title
BMKC_LS: Biomedical Knowledge Comprehension Last Sentence
CBT: Children's Book Test
CBT_NE: Children's Book Test Named Entity
CBT_Noun: Children's Book Test Noun Phrase
CNN: Cable News Network
GRU: Gated Recurrent Unit
ID: identification
MeSH: Medical Subject Headings
NLP: natural language processing
QA: question answering
