# Supplementary material for: A Pilot Study of Biomedical Text Comprehension using an Attention-Based Deep Neural Reader: Design and Experimental Analysis
Source: JMIR Med Inform. 2018 Jan 5;6(1):e2. doi: 10.2196/medinform.8751 (PMC5783222; doi:10.2196/medinform.8751)
Supplement: Multimedia Appendix 1 [file medinform_v6i1e2_app1.pdf]

## Multimedia Appendix 2

### Human evaluation details on the BMKC dataset

In this section, we report the responses of our machine comprehension model and the six human evaluators to the BMKC dataset.

Question 1.

| **Source dataset** | **BMKC_LS** |
|---|---|
| **Context** | Progranulin (PGRN) promotes cell growth and cell cycle progression in several cell types and contributes to tumorigenesis in diverse cancers. We have recently reported PGRN expression in islets and tumors developed in an MEN1 transgenic mouse. Here we sought to investigate PGRN expression and regulation after exposure to hypoxia as well as its effects on pancreatic islet cells and neuroendocrine tumors (NETs) in MEN1 mice. Gene and protein expression were analyzed by quantitative polymerase chain reaction, immunohistochemistry, and Western blot. We also investigated PGRN expression in samples from patients carrying pancreatic NETs associated or not with the multiple endocrine neoplasia 1 syndrome, using enzyme-linked immunosorbent assay and immunohistochemistry analysis. Progranulin is upregulated in tumors and islets of the MEN1 mouse as well as in the serum of patients with pancreatic NETs associated with glucagonoma syndrome. In normal mice islets and pancreatic tumors, PGRN expression was strongly potentiated by hypoxia. Progranulin promotes cell proliferation in islet cells and βTC-6 cells, a process paralleled by activation of the mitogen-activated protein kinase signaling cascade. |
| **Question** | Our findings identify PGRN as an effective inducer of pancreatic [........] proliferation and a possible important factor for pancreatic endocrine tumor development. |
| **Options** | carrying / cell proliferation / MEN1 / Western blot / cell / enzyme-linked immunosorbent assay / cell cycle / tumors / regulation / protein kinase / patients / mice / NETs / syndrome / polymerase chain reaction / glucagonoma syndrome / serum / islet cells / transgenic mouse / Gene / tumorigenesis / growth / hypoxia / multiple endocrine neoplasia / PGRN / immunohistochemistry / neuroendocrine tumors / |
| **Answer** | Islet cells |
| **Response** | |
| **CS undergrad 1** | Cell (x) |
| **CS undergrad 2** | Growth (x) |

| | |
|---|---|
| **Bio. Grad 1** | Islet cells (o) |
| **Bio. Grad 2** | Tumors (x) |
| **Expert 1** | Cell (x) |
| **Expert 2** | Islet cells (o) |
| **ASR model** | cells (x) |

Question 2.

| | |
|---|---|
| **Source dataset** | **BMKC_T** |
| **Context** | The lack of randomized clinical data pertaining to optimal surgery and adjuvant treatment in women with high-risk histotypes of endometrial cancer has resulted in selective management based on institutional policies. The objective of this study was to assess differences in treatment strategies and their outcomes among various institutions. High-risk endometrial cancer cases (2000-2012) with corresponding clinicopathologic data were collected from 7 academic cancer centers. Histotypes included grade 3 endometrioid (EC3), serous (ESC), clear cell (CCC) and carcinosarcoma (CS). Associations with overall survival were performed using Cox proportional hazard regression. 1260 patients treated between 2000 and 2012 were included in the study: 398 EC3, 449 ESC, 91 CCC, 236 CS and 83 'other'. The use of adjuvant chemotherapy, adjuvant radiation, and extent of surgical staging were statistically different among the 7 centers (P<0.001). Histotype was independently associated with overall survival (OS) in patients with stage 1 and 2 disease who underwent surgical staging (P=0.0324). Adjuvant radiation was associated with improved OS for EC3 and CCC and adjuvant chemotherapy was associated with improved OS for ESC and CS. There was a high rate of recurrence (17.8% and 21.4%) in completely staged, stage 1A patients with ESC and CS respectively. There exists a wide variation in practice and outcomes for high-risk histotypes of endometrial cancer. The relative impact of adjuvant therapy appears to be histotype dependent and prospective studies examining adjuvant treatment in high-risk histotypes should use caution combining them together. |
| **Question** | Treatment related outcomes in high-risk [........]: Canadian high risk endometrial cancer consortium (CHREC). |
| **Options** | adjuvant chemotherapy / institutional policies / cell / the 7 / Associations / risk / cancer / prospective studies / treatment / who / carcinosarcoma / recurrence / radiation / disease / patients / regression / endometrial cancer / survival / surgery / women / overall / |
| **Answer** | endometrial carcinoma |
| **Response** | |

| | |
|---|---|
| **CS undergrad 1** | endometrial cancer (o) |
| **CS undergrad 2** | endometrial cancer (o) |
| **Bio. Grad 1** | endometrial cancer (o) |
| **Bio. Grad 2** | endometrial cancer (o) |
| **Expert 1** | endometrial cancer (o) |
| **Expert 2** | endometrial cancer (o) |
| **ASR model** | endometrial cancer (o) |

Question 3.

| | |
|---|---|
| **Source dataset** | **BMKC_LS** |
| **Context** | Acromegaly is associated with increased thyroid cancer risk. We aimed to analyze the frequency of point mutations of BRAF and RAS genes, and RET/PTC, PAX8/PPARγ gene rearrangements in patients with acromegaly having differentiated thyroid cancers (DTC) and their relation with clinical and histological features. 14 acromegalic patients (8 male, 6 female) with DTC were included. BRAF V600E and NRAS codon 61 point mutations, RET/PTC1, RET/PTC3, and PAX8/PPARγ gene rearrangements were analyzed in thyroidectomy specimens. We selected 14 non-acromegalic patients with DTC as a control group. 2 patients (14.3%) were detected to have positive BRAF V600E and 3 patients (21.4%) were detected to have NRAS codon 61 mutation. NRAS codon 61 was the most frequent genetic alteration. Patients with positive mutation had aggressive histologic features more frequently than patients without mutations. Comparison of the acromegalic and non-acromegalic patients with DTC revealed that BRAF V600E mutation was more frequent in non-acromegalic patients with DTC (14.2% vs. 64.3%, p=0.02). RET/PTC 1/ 3, PAX8/PPARγ gene rearrangements were not detected in any patient. None of the patients including the patients with positive point mutations had recurrence, and local and/or distant metastasis. NRAS codon 61 is the most frequent genetic alteration in this acromegaly series with DTC. Since acromegalic patients have lower prevalance of BRAF V600E mutation, BRAF V600E mutation may not be a causative factor in development of DTC in acromegaly. |
| **Question** | Despite the relation of [........] V600E and NRAS codon 61 mutations with aggresive histopathologic features, their impact on tumor prognosis remains to be defined in acromegaly in further studies. |
| **Options** | point mutations / PTC / RAS genes / control group / PAX8 / PTC3 / RET / tumor / risk / NRAS / recurrence / female / male / |

| | |
|---|---|
| | acromegaly / codon / metastasis / patients / prognosis / thyroidectomy / mutation / gene rearrangements / thyroid cancers / BRAF / |
| **Answer** | BRAF |
| **Response** | |
| **CS undergrad 1** | BRAF (o) |
| **CS undergrad 2** | thyroid cancers (x) |
| **Bio. Grad 1** | BRAF (o) |
| **Bio. Grad 2** | BRAF(o) |
| **Expert 1** | BRAF(o) |
| **Expert 2** | BRAF(o) |
| **ASR model** | BRAF(o) |

Question 4.

| | |
|---|---|
| **Source dataset** | **BMKC_T** |
| **Context** | Previously, we developed a novel siRNA transfer method to the liver by sequential intravenous injection of anionic polymer and cationic liposome/siRNA complex (cationic lipoplex). In this study, we investigated whether siRNA delivered by this sequential injection could significantly suppress mRNA expression of the targeted gene in liver metastasis and inhibit tumor growth. When cationic lipoplex was intravenously injected into mice bearing liver metastasis of human breast tumor MCF-7 at 1 min after intravenous injection of chondroitin sulfate C (CS) or poly-l-glutamic acid (PGA), siRNA was accumulated in tumor-metastasized liver. In terms of a gene silencing effect, sequential injections of CS or PGA plus cationic lipoplex of luciferase siRNA could reduce luciferase activity in liver MCF-7-Luc metastasis. Regarding the side effects, sequential injections of CS plus cationic lipoplex did not exhibit hepatic damage or induction of inflammatory cytokines in serum after repeated injections, but sequential injections of PGA plus cationic lipoplex did. Finally, sequential injections of CS plus cationic lipoplex of protein kinase N3 siRNA could suppress tumor growth in the mice bearing liver metastasis. From these findings, sequential injection of CS and cationic lipoplex of siRNA might be a novel systemic method of delivering siRNA to liver metastasis. |
| **Question** | Therapeutic effect for liver-metastasized tumor by sequential [........] of anionic polymer and cationic lipoplex of siRNA. |
| **Options** | a gene / breast tumor / transfer / MCF-7 / injections / tumor / luciferase / liver / metastasis / mice / PGA / chondroitin sulfate C / cytokines / human / serum / gene / protein kinase N3 / growth |

| | |
|---|---|
| | / method / siRNA / polymer / intravenous injection / MCF-7-Luc / liposome / mRNA / |
| **Answer** | intravenous injection |
| **Response** | |
| **CS undergrad 1** | Injections (x) |
| **CS undergrad 2** | Injections (x) |
| **Bio. Grad 1** | Injections (x) |
| **Bio. Grad 2** | intravenous injection (o) |
| **Expert 1** | PGA (x) |
| **Expert 2** | Injections (x) |
| **ASR model** | Injection (x) |

Question 5.

| | |
|---|---|
| **Source dataset** | **BMKC_LS** |
| **Context** | The frequent development of drug resistance to targeted therapies in cancer patients has stimulated interest in strategies counteracting resistance. Combining immunotherapies with targeted therapies is one such strategy. In this context, we asked whether human NK cells can target melanoma cells that have acquired resistance to selective inhibitors targeting activating mutants of the B-Raf kinase (BRAF inhibitors, BRAFi). We generated drug-resistant cell variants in vitro from human BRAF-mutant melanoma cell lines MEL-HO, COLO-38, SK-MEL-37, 1520 and from primary melanoma cells freshly isolated from two patients. All drug-resistant cell variants remained susceptible to lysis by IL-2-activated NK cells; and two BRAFi-resistant lines (BRAFi-R) became significantly more susceptible to NK-cell lysis than their parental lines. This was associated with significant HLA class I antigen downregulation and PD-L1 upregulation on the drug-resistant lines. Although blocking HLA class I enhanced the extent of lysis of both BRAFi-R and parental cells to NK-cell-mediated lysis, antibody-mediated inhibition of PD1-PD-L1 interactions had no detectable effect. HLA class I antigen expression on BRAFi-R melanoma variants thus appears to play a major role in their susceptibility to NK-cell cytotoxicity. |
| **Question** | These findings suggest that NK-cell-based immunotherapy may be a viable approach to treat melanoma patients with acquired resistance to [........] inhibitors. |
| **Options** | role / cells / drug resistance / cancer / immunotherapies / inhibition / MEL-HO / patients / HLA class I antigen / downregulation / upregulation / PD-L1 / in vitro / human / BRAF inhibitors / SK-MEL-37 / melanoma / NK-cell / PD1 / |

| | |
|---|---|
| | COLO-38 / IL-2 / kinase / B-Raf / |
| **Answer** | BRAF |
| **Response** | |
| **CS undergrad 1** | B-Raf (o) |
| **CS undergrad 2** | B-Raf (o) |
| **Bio. Grad 1** | B-Raf (o) |
| **Bio. Grad 2** | B-Raf (o) |
| **Expert 1** | BRAF(o) |
| **Expert 2** | B-Raf (o) |
| **ASR model** | BRAF(o) |

Question 6.

| | |
|---|---|
| **Source dataset** | **BMKC_T** |
| **Context** | The LNCaP cell line was originally isolated from the lymph node of a patient with metastatic prostate cancer. Many cell lines have been derived from LNCaP by selective pressures to study different aspects of prostate cancer progression. When injected subcutaneously into male athymic nude mice, LNCaP and its derivatives rarely metastasize. Here, we describe the characteristics of a new LNCaP derivative, JHU-LNCaP-SM, which was generated by long term passage in normal cell culture conditions. Short tandem repeat (STR) analysis and genomic sequencing verified JHU-LNCaP-SM derivation from parental LNCaP cells. JHU-LNCaP-SM cells express the same mutated androgen receptor (AR) but unlike LNCaP, are no longer androgen dependent for growth. The cells demonstrate an attenuated androgen responsiveness in transcriptional assays and retain androgen sensitive expression of PSA, AR, and PSMA. Unlike parental LNCaP, JHU-LNCaP-SM cells quickly form subcutaneous tumors in male athymic nude mice, reliably metastasize to the lymph nodes and display a striking intra-tumoral and spreading hemorrhagic phenotype as tumor xenografts. The JHU-LNCaP-SM cell line is a new isolate of LNCaP, which facilitates practical, preclinical studies of spontaneous metastasis of prostate cancer through lymphatic tissues. Prostate 76:215-225, 2016. © 2015 Wiley Periodicals, Inc. |
| **Question** | Characterization of a novel metastatic [........] cell line of LNCaP origin. |
| **Options** | LNCaP / nude mice / cells / tumor / male / lymph node / cell culture / PSA / metastasis / patient / prostate cancer / PSMA / pressures / phenotype / xenografts / Short tandem repeat / AR / Periodicals / growth / androgen receptor / cell line / STR / |

| | |
|---|---|
| | lymphatic tissues / Prostate / |
| **Answer** | prostate cancer |
| **Response** | |
| **CS undergrad 1** | prostate cancer (o) |
| **CS undergrad 2** | prostate cancer (o) |
| **Bio. Grad 1** | prostate cancer (o) |
| **Bio. Grad 2** | prostate cancer (o) |
| **Expert 1** | prostate cancer (o) |
| **Expert 2** | lymph node (x) |
| **ASR model** | prostate cancer (o) |

Question 7.

| | |
|---|---|
| **Source dataset** | **BMKC_LS** |
| **Context** | The Breast Cancer Risk Assessment Tool (BCRAT, "Gail model") is commonly used for breast cancer prediction; however, it has not been validated for women age 75 years and older. We used Nurses' Health Study (NHS) data beginning in 2004 and Women's Health Initiative (WHI) data beginning in 2005 to compare BCRAT's performance among women age 75 years and older with that in women age 55 to 74 years in predicting five-year breast cancer incidence. BCRAT risk factors include: age, race/ethnicity, age at menarche, age at first birth, family history, history of benign breast biopsy, and atypia. We examined BCRAT's calibration by age by comparing expected/observed (E/O) ratios of breast cancer incidence. We examined discrimination by computing c-statistics for the model by age. All statistical tests were two-sided. Seventy-three thousand seventy-two NHS and 97 081 WHI women participated. NHS participants were more likely to be non-Hispanic white (96.2% vs 84.7% in WHI, P < .001) and were less likely to develop breast cancer (1.8% vs 2.0%, P = .02). E/O ratios by age in NHS were 1.16 (95% confidence interval [CI] = 1.09 to 1.23, age 57-74 years) and 1.31 (95% CI = 1.18 to 1.45, age ≥ 75 years, P = .02), and in WHI 1.03 (95% CI = 0.97 to 1.09, age 55-74 years) and 1.10 (95% CI = 1.00 to 1.21, age ≥ 75 years, P = .21). E/O ratio 95% confidence intervals crossed one among women age 75 years and older when samples were limited to women who underwent mammography and were without significant illness. C-statistics ranged between 0.56 and 0.58 in both cohorts regardless of age. BCRAT accurately predicted breast cancer for women age 75 years and older who underwent mammography and were without significant illness but had modest discrimination. |

| | |
|---|---|
| **Question** | Models that consider individual competing risks of non-breast cancer death may improve [........] risk prediction for older women. |
| **Options** | breast cancer / first birth / Women's Health / confidence intervals / biopsy / death / calibration / Nurses / breast / statistics / risk / risk factors / Hispanic / who / discrimination / menarche / incidence / white / Risk Assessment / NHS / mammography / race / history / Health / family / women / |
| **Answer** | breast cancer |
| **Response** | |
| **CS undergrad 1** | breast cancer (o) |
| **CS undergrad 2** | mammography (x) |
| **Bio. Grad 1** | breast cancer (o) |
| **Bio. Grad 2** | breast cancer (o) |
| **Expert 1** | breast cancer (o) |
| **Expert 2** | incidence (x) |
| **ASR model** | breast cancer (o) |

Question 8.

| | |
|---|---|
| **Source dataset** | **BMKC_T** |
| **Context** | Carbonyl reductase (CBR) catalyzes anthracycline metabolism, and single nucleotide polymorphisms (SNPs) in CBR impact metabolic efficiency. In pediatric patients, homozygosity for the major allele (G) in the CBR3 gene was associated with increased risk of anthracycline cardiotoxicity. We hypothesized that CBR SNPs contribute to cardiotoxicity in adults. We retrospectively identified female breast cancer patients in the Columbus Breast Tissue Bank Registry treated with adriamycin and cytoxan (AC) from 2003 to 2012. We selected patients who developed cardiomyopathy, defined as a drop in ejection fraction to $<50$ % or $>15$ % decrease from pre-therapy. Univariate and multivariate logistic regressions were performed to identify cardiotoxicity risk factors. SNPs were genotyped, and frequency of the major allele (G)/minor allele (A) of the CBR3 and CBR1 genes was calculated. We identified 52 cases of cardiotoxicity after AC and 110 controls. Multivariate analysis showed that trastuzumab ($p = 0.009$), diabetes ($p = 0.05$), and consumption of $>8$ alcoholic drinks/week ($p = 0.024$) were associated with higher cardiotoxicity risk. Moderate alcohol consumption ($<8$ drinks/week) was associated with lower risk ($p = 0.009$). No association was identified between CBR SNPs and cardiotoxicity (CBR1 $p = 0.261$; CBR3 $p = 0.556$). This is the first study to evaluate SNPs in the CBR pathway as predictors of AC cardiotoxicity in adults. We did not observe |

| | |
|---|---|
| | any significant correlation between cardiotoxicity and SNPs within the CBR pathway. Further investigation into CBR SNPs in a larger adult sample is needed. Additional exploration into genomic predictors of anthracycline cardiotoxicity may allow for the development of preventative and therapeutic strategies for those at risk. |
| **Question** | Risk factors for anthracycline-associated [........]. |
| **Options** | SNPs / breast cancer / Registry / cardiotoxicity / association / adriamycin / Breast / cytoxan / risk / risk factors / cardiomyopathy / who / therapeutic / female / minor / patients / CBR1 / reductase / CBR3 / Multivariate analysis / adults / alcohol consumption / trastuzumab / allele / efficiency / consumption / metabolism / Tissue Bank / logistic regressions / alcoholic / |
| **Answer** | cardiotoxicity |
| **Response** | |
| **CS undergrad 1** | cardiotoxicity (o) |
| **CS undergrad 2** | CBR3 (x) |
| **Bio. Grad 1** | CBR3 (x) |
| **Bio. Grad 2** | cardiotoxicity (o) |
| **Expert 1** | SNPs (x) |
| **Expert 2** | cardiotoxicity (o) |
| **ASR model** | cardiotoxicity (o) |

Question 9.

| | |
|---|---|
| **Source dataset** | **BMKC_LS** |
| **Context** | Glioblastoma multiforme (GBM) are the most common primary malignant brain tumor in adults, with a median survival of about one year. This poor prognosis is attributed primarily to therapeutic resistance and tumor recurrence after surgical removal, with the root cause suggested to be found in glioblastoma stem cells (GSCs). Using glial fibrillary acidic protein (GFAP) as a reporter of astrocytic differentiation, we isolated multiple clones from three independent GSC lines which express GFAP in a remarkably stable fashion. We next show that elevated expression of GFAP is associated with reduced clonogenicity in vitro and tumorigenicity in vivo. Utilizing this in vitro cell-based differentiation reporter system we screened chemical libraries and identified the non-depolarizing neuromuscular blocker (NNMB), Atracurium Besylate, as a small molecule which effectively induces astroglial but not neuronal differentiation of GSCs. Functionally, Atracurium Besylate treatment significantly |

| | |
|---|---|
| | inhibited the clonogenic capacity of several independent patient-derived GSC neurosphere lines, a phenomenon which was largely irreversible. A second NNMB, Vecuronium, also induced GSC astrocytic differentiation while Dimethylphenylpiperazinium (DMPP), a nicotinic acetylcholine receptor (nAChR) agonist, significantly blocked Atracurium Besylate pro-differentiation activity. To investigate the clinical importance of nAChRs in gliomas, we examined clinical outcomes and found that glioma patients with tumors overexpressing CHRNA1 or CHRNA9 (encoding for the AChR-α1 or AChR-α9) exhibit significant shorter overall survival. |
| **Question** | Finally, we found that ex-vivo pre-treatment of GSCs, expressing [........] and CHRNA9, with Atracurium Besylate significantly increased the survival of mice xenotransplanted with these cells, therefore suggesting that tumor initiating subpopulations have been reduced. |
| **Options** | Glioblastoma multiforme / clones / glial fibrillary acidic protein / cells / CHRNA9 / GFAP / tumors / therapeutic / recurrence / nicotinic acetylcholine receptor / CHRNA1 / patient / stem cells / mice / survival / prognosis / overall / adults / GSC / in vitro / brain tumor / gliomas / Atracurium Besylate / DMPP / Vecuronium / chemical libraries / |
| **Answer** | CHRNA1 |
| **Response** | |
| **CS undergrad 1** | CHRNA1 (o) |
| **CS undergrad 2** | prognosis (x) |
| **Bio. Grad 1** | CHRNA1 (o) |
| **Bio. Grad 2** | CHRNA1 (o) |
| **Expert 1** | CHRNA1 (o) |
| **Expert 2** | glial fibrillary acidic protein (x) |
| **ASR model** | CHRNA1 (o) |

Question 10.

| | |
|---|---|
| **Source dataset** | **BMKC_T** |
| **Context** | Signal transducer and activator of transcription (STAT) 3 is a key factor in multiple tyrosine kinase inhibitor (mTKI)-induced growth inhibition and apoptosis of renal cell carcinoma (RCC) cells. This study aimed to identify associations between single-nucleotide polymorphisms (SNPs) in the STAT3 gene and tumor response to mTKIs in patients with metastatic RCC (mRCC). Seventy-one patients with clear cell RCC treated with any mTKI were retrospectively genotyped to elucidate a potential |

| | |
|---|---|
| | association between STAT3 SNPs and overall best response to drugs. Of 50 patients included for analysis, a partial or complete response was observed in 17. A significant association was found between rs4796793 alleles and tumor response [G vs. C, odds ratio (OR) 3.25, 95 % confidence interval (CI) 1.30-8.07]. There were a higher percentage of responders with the C/C genotype at rs4796793 than with the G/C + G/G genotypes (OR 4.46, 95 % CI 1.31-15.28). Time-to-event analysis demonstrated a statistically significant difference between patients with the CC genotype and those with G/C + G/G genotypes in time-to-treatment response, but not in progression-free survival or time-to-treatment failure. The rs4796793 genotype is a novel predictive factor of the response to mTKIs in patients with mRCC. However, prospective translational trials with larger patient cohorts are required to confirm these results. |
| **Question** | STAT3 polymorphism rs4796793 may be a predictive factor of tumor response to multiple [........] inhibitors in metastatic renal cell carcinoma in Japanese population. |
| **Options** | SNPs / CI 1 / confidence interval / association / mRCC / cell / tumor / C/C / renal cell carcinoma / patients / STAT3 / Time / overall / genotype / progression-free survival / alleles / time-to-treatment / gene / growth / STAT / drugs / tyrosine kinase / odds ratio / apoptosis / RCC / |
| **Answer** | tyrosine kinase |
| **Response** | |
| **CS undergrad 1** | tyrosine kinase (o) |
| **CS undergrad 2** | mRCC (x) |
| **Bio. Grad 1** | tyrosine kinase (o) |
| **Bio. Grad 2** | tyrosine kinase (o) |
| **Expert 1** | tyrosine kinase (o) |
| **Expert 2** | tyrosine kinase (o) |
| **ASR model** | tyrosine kinase (o) |

Question 11.

| | |
|---|---|
| **Source dataset** | **BMKC_LS** |
| **Context** | A recent randomized radiation dose-escalation trial in unresectable stage III non-small-cell lung cancer (NSCLC) (Radiation Therapy Oncology Group [RTOG] 0617) showed a lower survival rate in the high-dose radiation therapy (RT) arm (74 Gy) than in the low-dose arm (60 Gy) with concurrent chemotherapy. The primary QOL hypothesis predicted a clinically meaningful decline in quality of life (QOL) via the Functional |

| | |
|---|---|
| | Assessment of Cancer Therapy (FACT)-Lung Cancer Subscale (LCS) in the high-dose RT arm at 3 months. The RTOG 0617 trial was a randomized phase 3 study (conducted from November 2007 to November 2011) in stage III NSCLC using a 2 × 2 factorial design and stratified by histology, positron emission tomography staging, performance status, and irradiation technique (3-dimensional conformal RT [3D-CRT] vs intensity-modulated RT [IMRT]). A total of 185 institutions in the United States and Canada took part. Of 424 eligible patients with stage III NSCLC randomized, 360 (85%) consented to QOL evaluation, of whom 313 (88%) completed baseline QOL assessments. Treatment with 74-Gy vs 60-Gy RT with concurrent and consolidation carboplatin/paclitaxel with or without cetuximab. The QOL data were collected prospectively via FACT Trial Outcome Index (FACT-TOI), calculated as the sum of the following measures: Physical Well Being (PWB), Functional Well Being (FWB), and the LCS. Data are presented at baseline and 3 and 12 months via minimal clinically meaningful changes of 2 points or more for PWB, FWB, and LCS or 5 points or more for TOI. Of the 313 patients who completed baseline QOL assessments, 219 patients (70%) completed the 3-month QOL assessments, and 137 of the living patients (57%) completed the 12-month assessment. Patient demographics and baseline QOL scores were comparable between the 74-Gy and 60-Gy arms. Significantly more patients in the 74-Gy arm than in the 60-Gy arm had clinically meaningful decline in FACT-LCS at 3 months (45% vs 30%; P = .02). At 12 months, fewer patients who received IMRT (vs 3D-CRT) had clinically meaningful decline in FACT-LCS (21% vs 46%; P = .003). Baseline FACT-TOI was associated with overall survival in multivariate analysis. Despite few differences in clinician-reported toxic effects between treatment arms, QOL analysis demonstrated a clinically meaningful decline in QOL in the 74-Gy arm at 3 months, confirming the primary QOL hypothesis. |
| **Question** | Baseline QOL was an independent prognostic factor for [........]. clinicaltrials.gov Identifier: NCT00533949. |
| **Options** | positron emission tomography / United States / chemotherapy / evaluation / histology / Cancer / who / Treatment / Canada / Lung Cancer / patients / multivariate analysis / survival / survival rate / overall / CRT / LCS / cetuximab / carboplatin/paclitaxel / demographics / arm / NSCLC / FACT / non-small-cell lung cancer / quality of life / radiation / measures / |
| **Answer** | survival |
| **Response** | |
| **CS** | non-small-cell lung cancer (x) |

| | |
|---|---|
| **undergrad 1** | |
| **CS undergrad 2** | demographics (x) |
| **Bio. Grad 1** | FACT (x) |
| **Bio. Grad 2** | non-small-cell lung cancer (x) |
| **Expert 1** | NSCLC (x) |
| **Expert 2** | radiation (x) |
| **ASR model** | Survival (o) |

Question 12.

| | |
|---|---|
| **Source dataset** | **BMKC_T** |
| **Context** | The Wnt inhibitor Dickkopf-1 (DKK-1) has been associated with the occurrence of bone metastases in osteotropic prostate cancer by inhibiting osteoblastogenesis. P38 mitogen-activated protein kinase (MAPK) activity is also dysregulated in advanced prostate cancer. However, the impact of p38 MAPK signaling on DKK-1 remains unknown. Inhibition of p38 MAPK signaling in osteolytic PC3 cells by small molecule inhibitors (doramapimod, LY2228820 and SB202190) suppressed DKK-1 expression, whereas activation of p38 MAPK by anisomycin increased DKK-1. Further dissection by targeting individual p38 MAPK isoforms with siRNA revealed a stronger role for MAPK11 than MAPK14 and MAPK12 in the regulation of DKK-1. Moreover, prostate cancer cells with a predominantly osteolytic phenotype produced sufficient amounts of DKK-1 to inhibit Wnt3a-induced osteoblastic differentiation in C2C12 cells. This inhibition was blocked directly by neutralizing DKK-1 using a specific antibody and also indirectly by blocking p38 MAPK. Furthermore, tissue expression in human prostate cancer revealed a correlation between p38 MAPK and DKK-1 expression with higher expression in tumor compared with normal tissues. These results reveal that p38 MAPK regulates DKK-1 in prostate cancer and may present a potential target in osteolytic prostate cancers. |
| **Question** | p38 MAPK regulates the Wnt inhibitor Dickkopf-1 in osteotropic prostate cancer [........]. |
| **Options** | MAPK12 / role / cells / bone / DKK-1 / tumor / inhibition / dissection / regulation / metastases / prostate cancer / tissue / doramapimod / Dickkopf-1 / p38 MAPK / phenotype / isoforms / PC3 / MAPK11 / human / anisomycin / P38 mitogen-activated protein kinase / MAPK14 / siRNA / Wnt3a / |
| **Answer** | cells |
| **Response** | |
| **CS undergrad 1** | bone (x) |

| | |
|---|---|
| **CS undergrad 2** | siRNA (x) |
| **Bio. Grad 1** | metastases (x) |
| **Bio. Grad 2** | cells (o) |
| **Expert 1** | cells (o) |
| **Expert 2** | metastases(x) |
| **ASR model** | Cells (o) |

Question 13.

| | |
|---|---|
| **Source dataset** | **BMKC_LS** |
| **Context** | A multianalyte chemiluminescence (CL) imaging immunoassay strategy for sensitive detection of different isoforms of prostate specific antigen (PSA) was developed. The microtiter plates were fabricated by simultaneously immobilizing of free-PSA (f-PSA) and total-PSA (t-PSA) capture antibody on nitrocellulose (NC) membrane. Each of the array were spotted in replicates of six spots within a spacing of 2mm. 16 or 48 detection wells were integrated on a single NC membrane and each well could be used as a microreactor and microanalysis chamber. Under a sandwiched immunoassay, the CL signals on each sensing site were collected by a charge-coupled device (CCD), presenting an array-based chemiluminescence imaging. Soybean peroxidase (SBP) was used to label f-PSA or t-PSA monoclonal antibody. With the amplification effects of two enhancers, 3-(10'-phenothiazinyl) propane-1-sulfonate (SPTZ) and 4-morpholinopyridine (MORP), the CL intensity could significantly enhanced, which improved the sensing sensitivity and detection limit. Under the optimal conditions, the linear response to the analyte concentration ranged from 0.01-36.7ng/mL and 0.02-125ng/mL for f-PSA and t-PSA, respectively. The results for the detection of forty serum samples from prostate cancer patients and cancer-free patients showed good agreement with the clinical data, suggesting that the proposed assay had acceptable accuracy. |
| **Question** | The proposed CL imaging immunoassay possess high throughput and acceptable reproducibility, stability and accuracy, which made it great potential to available to distinguish different isoforms of PSA in [........] samples. |
| **Options** | prostate specific antigen / chemiluminescence / 4-morpholinopyridine / sensitivity / concentration / charge / detection limit / nitrocellulose / cancer / SBP / PSA / CCD / patients / prostate cancer / isoforms / device / serum / immunoassay / propane-1-sulfonate / membrane / Soybean / peroxidase / |
| **Answer** | Serum |

| **Response** | |
|---|---|
| **CS undergrad 1** | Serum (o) |
| **CS undergrad 2** | SBP (x) |
| **Bio. Grad 1** | prostate cancer (x) |
| **Bio. Grad 2** | prostate specific antigen (x) |
| **Expert 1** | prostate cancer (x) |
| **Expert 2** | Serum (o) |
| **ASR model** | Serum (o) |

Question 14.

| **Source dataset** | **BMKC_T** |
|---|---|
| **Context** | Peritoneal dissemination of gastric cancer is still a dismal disease and has extremely poor prognosis even with systemic intensive chemotherapy. However, intraperitoneal chemotherapy using paclitaxel has recently shown good results. In order to perform optimal intraperitoneal chemotherapy, laparoscopic examination is necessary to assess the condition of peritoneal disseminated lesions. This is the first report of a case of a patient with gastric cancer with massive peritoneal metastasis treated with intraperitoneal administration of paclitaxel and repeated laparoscopic examinations who survived more than 5 years. Here we report a case of a 60-year-old Japanese woman with peritoneal carcinomatosis of gastric cancer who underwent intraperitoneal chemotherapy receiving repeated laparoscopic examinations. The patient was referred to our institution for the treatment of peritoneal carcinomatosis of gastric cancer. The staging laparoscopy showed peritoneal metastasis in the whole peritoneal space with a peritoneal cancer index score of 23. An intraperitoneal access port was subcutaneously implanted. Paclitaxel was intraperitoneally and intravenously administered with oral administration of S-1. The second-look laparoscopy, which was performed after nine courses of intraperitoneal chemotherapy, revealed the disappearance of peritoneal carcinomatosis. A total gastrectomy with D2 lymphadenectomy was performed and intraperitoneal chemotherapy was continued after the surgery. The third laparoscopic examination, which was performed after 67 courses of intraperitoneal chemotherapy showed bilateral ovarian metastasis without recurrence of peritoneal carcinomatosis. Since multiple bone metastases developed after the third-look laparoscopy, bilateral adnexectomy was not performed and the chemotherapy was changed to the regimen including CPT-11. Our patient survived more than 5 |

| | |
|---|---|
| | years since the intraperitoneal chemotherapy started. Sequential intraperitoneal chemotherapy could strongly suppress the development of peritoneal metastasis for several years. Repeated laparoscopic examinations are considered to be essential to evaluate the efficacy of intraperitoneal chemotherapy on peritoneal carcinomatosis of gastric cancer. |
| **Question** | A [........] with gastric cancer with peritoneal carcinomatosis treated with intraperitoneal chemotherapy who survived more than 5 years receiving repeated laparoscopic examinations: a case report. |
| **Options** | |
| **Answer** | Patient |
| **Response** | gastric cancer / chemotherapy / bone / carcinomatosis / gastrectomy / report / cancer / who / recurrence / treatment / disease / metastases / lymphadenectomy / paclitaxel / patient / prognosis / Japanese / oral administration / administration / laparoscopy / CPT-11 / surgery / woman / |
| **CS undergrad 1** | report (x) |
| **CS undergrad 2** | Patient (o) |
| **Bio. Grad 1** | Woman (x) |
| **Bio. Grad 2** | patient (o) |
| **Expert 1** | metastases (x) |
| **Expert 2** | metastases (x) |
| **ASR model** | Patient (o) |

Question 15.

| | |
|---|---|
| **Source dataset** | **BMKC_LS** |
| **Context** | Optimally effective antitumor therapies would not only activate immune effector cells but also engage them at the tumor. Folate conjugated to immunoglobulin (F-IgG) could direct innate immune cells with Fc receptors to folate receptor-expressing cancer cells. F-IgG bound to human KB and HeLa cells, as well as murine L1210JF, a folate receptor (FR)-overexpressing cancer cell line, as determined by flow cytometry. Recognition of F-IgG by natural killer (NK) cell Fc receptors led to phosphorylation of the ERK transcription factor and increased NK cell expression of CD69. Lysis of KB tumor cells by NK cells increased by about 5-fold after treatment with F-IgG, an effect synergistically enhanced by treatment with IL2, IL12, IL15, or IL21 ($P < 0.001$). F-IgG also enhanced the lysis of chronic lymphocytic leukemia cells by autologous NK cells. NK cells significantly increased production of IFNγ, MIP-1α, and RANTES in response to F-IgG-coated KB target |

| | |
|---|---|
| | cells in the presence of the NK cell-activating cytokine IL12, and these coculture supernatants induced significant T-cell chemotaxis (P< 0.001). F-IgG-coated targets also stimulated FcR-mediated monocyte effector functions. Studies in a murine leukemia model confirmed the intratumoral localization and antitumor activity of F-IgG, as well as enhancement of its effects by IL12 (P =0.05). |
| **Question** | The antitumor effect of this combination was dependent on NK cells and led to decreased tumor cell proliferationin vivo Thus, F-IgG can induce an immune response against FR-positive [........] cells that is mediated by NK cells and can be augmented by cytokine therapy.Cancer Immunol Res; 4(4); 323-36. ©2016 AACR. |
| **Options** | after treatment / IL12 / chronic lymphocytic leukemia / cell / MIP / chemotaxis / tumor / treatment / phosphorylation / monocyte / ERK / coculture / leukemia / human / RANTES / HeLa cells / production / folate receptor / flow cytometry / immunoglobulin / NK cells / IL2 / IgG / Folate / transcription factor / IL15 / IL21 / CD69 / T-cell / Fc receptors / |
| **Answer** | Tumor |
| **Response** | |
| **CS undergrad 1** | Tumor |
| **CS undergrad 2** | IL12 (x) |
| **Bio. Grad 1** | Tumor (o) |
| **Bio. Grad 2** | Tumor (o) |
| **Expert 1** | leukemia (x) |
| **Expert 2** | chronic lymphocytic leukemia (x) |
| **ASR model** | Tumor (o) |

Question 16.

| | |
|---|---|
| **Source dataset** | **BMKC_T** |
| **Context** | Targeting the TGF-β1 pathway for breast cancer metastasis therapy has become an attractive strategy. We have previously demonstrated that naringenin significantly reduced TGF-β1 levels in bleomycin-induced lung fibrosis and effectively prevented pulmonary metastases of tumors. This raised the question of whether naringenin can block TGF-β1 secretion from breast cancer cells and inhibit their pulmonary metastasis. We transduced a lentiviral vector encoding the mouse Tgf-β1 gene into mouse breast carcinoma (4T1-Luc2) cells and inoculated the transformant cells (4T1/TGF-β1) into the fourth primary fat pat of Balb/c mice. Pulmonary metastases derived from the primary |

| | |
|---|---|
| | tumors were monitored using bioluminescent imaging. Spleens, lungs and serum (n = 18-20 per treatment group) were analyzed for immune cell activity and TGF-β1 level. The mechanism whereby naringenin decreases TGF-β1 secretion from breast cancer cells was investigated at different levels, including Tgf-β1 transcription, mRNA stability, translation, and extracellular release. In contrast to the null-vector control (4T1/RFP) tumors, extensive pulmonary metastases derived from 4T1/TGF-β1 tumors were observed. Administration of the TGF-β1 blocking antibody 1D11 or naringenin showed an inhibition of pulmonary metastasis for both 4T1/TGF-β1 tumors and 4T1/RFP tumors, resulting in increased survival of the mice. Compared with 4T1/RFP bearing mice, systemic immunosuppression in 4T1/TGF-β1 bearing mice was observed, represented by a higher proportion of regulatory T cells and myeloid-derived suppressor cells and a lower proportion of activated T cells and INFγ expression in CD8(+) T cells. These metrics were improved by administration of 1D11 or naringenin. However, compared with 1D11, which neutralized secreted TGF-β1 but did not affect intracellular TGF-β1 levels, naringenin reduced the secretion of TGF-β1 from the cells, leading to an accumulation of intracellular TGF-β1. Further experiments revealed that naringenin had no effect on Tgf-β1 transcription, mRNA decay or protein translation, but prevented TGF-β1 transport from the trans-Golgi network by inhibiting PKC activity. Naringenin blocks TGF-β1 trafficking from the trans-Golgi network by suppressing PKC activity, resulting in a reduction of TGF-β1 secretion from breast cancer cells. This finding suggests that naringenin may be an attractive therapeutic candidate for TGF-β1 related diseases. |
| **Question** | [........] prevents TGF-β1 secretion from breast cancer and suppresses pulmonary metastasis by inhibiting PKC activation. |
| **Options** | affect / CD8 / breast cancer / cells / bleomycin / Tgf / tumors / therapy / inhibition / metrics / PKC / immunosuppression / diseases / metastases / mice / naringenin / Spleens / survival / mRNA stability / protein translation / Balb/c mice / serum / administration / gene / trans-Golgi network / fibrosis / secretion / lungs / regulatory T cells / T cells / RFP / |
| **Answer** | Naringenin |
| **Response** | |
| **CS undergrad 1** | trans-Golgi network (x) |
| **CS undergrad 2** | naringenin (o) |
| **Bio. Grad 1** | naringenin (o) |
| **Bio. Grad 2** | naringenin (o) |

| | |
|---|---|
| **Expert 1** | naringenin (o) |
| **Expert 2** | naringenin (o) |
| **ASR model** | naringenin (o) |

Question 17.

| | |
|---|---|
| **Source dataset** | **BMKC_LS** |
| **Context** | Myxopapillary ependymomas (MPEs) are rare spinal tumors in children. The natural history and clinical course of pediatric MPEs are largely unknown and the indication for adjuvant therapy remains to be clarified. We performed an IRB-approved, retrospective review of children with MPEs treated at the Dana-Farber/Boston Children's Cancer and Blood Disorder Center between 1982 and 2013. Eighteen children (age range 8-21 years, median age 14 years) met inclusion criteria. We reviewed the histopathology, magnetic resonance imaging, tumor location and stage, surgical management, adjuvant therapy, and clinical outcomes. The median follow-up duration was 9.4 years (range 1-30 years). Children most commonly presented with pain, scoliosis, and urinary symptoms. All primary tumors were located in the lower thoracic or lumbar spine. Nine children (50%) had leptomeningeal tumor seeding at presentation, most commonly located within the distal thecal sac. A gross-total resection was achieved in nine children (50%). Three children were treated with irradiation following initial surgery. No child received adjuvant chemotherapy at diagnosis. The 10-year event-free survival (EFS) was 26% ± 14.8. Children with disseminated disease trended towards inferior EFS compared to those with localized disease (10-year EFS 12.7% ± 12 vs. 57 ± 25%, p value 0.07). The 10-year overall survival was 100%. The efficacy of adjuvant irradiation could not be assessed due to the small sample size. Although children with MPEs frequently present with disseminated tumor and/or develop recurrent or progressive disease, their overall survival is excellent. |
| **Question** | Treatment should aim to minimize both [........]- and therapy-related morbidity. |
| **Options** | Blood / Boston / spine / IRB / tumor / magnetic resonance imaging / therapy / disease / survival / sample size / scoliosis / overall / pain / Myxopapillary ependymomas / adjuvant chemotherapy / diagnosis / natural history / children / event-free survival / Farber / EFS / morbidity / review / surgery / |
| **Answer** | Tumor |
| **Response** | |
| **CS undergrad 1** | magnetic resonance imaging (x) |

| | |
|---|---|
| **CS undergrad 2** | chemotherapy (x) |
| **Bio. Grad 1** | event-free survival (x) |
| **Bio. Grad 2** | surgery (x) |
| **Expert 1** | Tumor (o) |
| **Expert 2** | disease (x) |
| **ASR model** | Tumor (o) |

Question 18.

| | |
|---|---|
| **Source dataset** | **BMKC_T** |
| **Context** | Trastuzumab resistance is a key therapeutic challenge in metastatic breast cancer. We postulated that broader inhibition of ErbB receptors with afatinib would improve clinical outcomes compared with HER2 inhibition alone in patients who had progressed on previous trastuzumab treatment. LUX-Breast 1 compared afatinib plus vinorelbine with trastuzumab plus vinorelbine for such patients with HER2-positive metastatic breast cancer. We did this open-label trial at 350 hospitals in 41 countries worldwide. We enrolled female patients with HER2-overexpressing metastatic breast cancer who had progressed on or following adjuvant trastuzumab or first-line treatment of metastatic disease with trastuzumab. Participants were randomly assigned (2:1) to receive oral afatinib (40 mg/day) plus intravenous vinorelbine (25 mg/m(2) per week) or intravenous trastuzumab (2 mg/kg per week after 4 mg/kg loading dose) plus vinorelbine. Randomisation was done centrally and stratified by previous trastuzumab treatment (adjuvant vs first-line treatment), hormone receptor status (oestrogen receptor and progesterone receptor positive vs others), and region. The primary endpoint was progression-free survival, assessed in the intention-to-treat population. This trial is closed to enrolment and is registered with ClinicalTrials.gov, NCT01125566. Between Aug 26, 2010, and April 26, 2013, we enrolled 508 patients: 339 assigned to the afatinib group and 169 assigned to the trastuzumab group. Recruitment was stopped on April 26, 2013, after a benefit-risk assessment by the independent data monitoring committee was unfavourable for the afatinib group. Patients on afatinib plus vinorelbine had to switch to trastuzumab plus vinorelbine, afatinib monotherapy, vinorelbine monotherapy, or receive treatment outside of the trial. Median follow-up was 9·3 months (IQR 3·7-16·0). Median progression-free survival was 5·5 months (95% CI 5·4-5·6) in the afatinib group and 5·6 months (5·3-7·3) in the trastuzumab group (hazard ratio 1·10 95% CI 0·86-1·41; p=0·43). The most common drug-related |

| | |
|---|---|
| | adverse events of grade 3 or higher were neutropenia (190 [56%] of 337 patients in the afatinib group vs 102 [60%] of 169 patients in the trastuzumab group), leucopenia (64 [19%] vs 34 [20%]), and diarrhoea (60 [18%] vs none). Trastuzumab-based therapy remains the treatment of choice for patients with HER2-positive metastatic breast cancer who had progressed on trastuzumab. Boehringer Ingelheim. |
| **Question** | Afatinib plus vinorelbine versus trastuzumab plus vinorelbine in patients with HER2-overexpressing metastatic [........] who had progressed on one previous trastuzumab treatment (LUX-Breast 1): an open-label, randomised, phase 3 trial. |
| **Options** | breast cancer / HER2 / ErbB receptors / oestrogen receptor / intention / Breast / neutropenia / who / treatment / inhibition / female / data monitoring committee / disease / population / vinorelbine / patients / hormone / benefit-risk assessment / afatinib / progression-free survival / Trastuzumab / hospitals / progesterone receptor / |
| **Answer** | breast cancer |
| **Response** | |
| **CS undergrad 1** | breast cancer (o) |
| **CS undergrad 2** | breast cancer (o) |
| **Bio. Grad 1** | breast cancer (o) |
| **Bio. Grad 2** | breast cancer (o) |
| **Expert 1** | breast cancer (o) |
| **Expert 2** | breast cancer (o) |
| **ASR model** | breast cancer (o) |

Question 19.

| | |
|---|---|
| **Source dataset** | **BMKC_LS** |
| **Context** | Autologous adipose tissue transfer may be performed for aesthetic needs following resection of osteosarcoma, the most frequent primary malignant tumor of bone, excluding myeloma. The safety of autologous adipose tissue transfer regarding the potential risk of cancer recurrence must be addressed. Adipose tissue injection was tested in a human osteosarcoma preclinical model induced by MNNG-HOS cells. Culture media without growth factors from fetal bovine serum were conditioned with adipose tissue samples and added to two osteosarcoma cell lines (MNNG-HOS and MG-63) that were cultured in monolayer or maintained in nonadherent spheres, favoring a proliferation or quiescent stage, respectively. Proliferation and cell cycle were analyzed. Adipose tissue injection increased local growth of |

| | |
|---|---|
| | osteosarcoma in mice but was not associated with aggravation of lung metastasis or osteolysis. Adipose tissue-derived soluble factors increased the in vitro proliferation of osteosarcoma cells up to 180 percent. Interleukin-6 and leptin were measured in higher concentrations in adipose tissue-conditioned medium than in osteosarcoma cell-conditioned medium, but the authors' results indicated that they were not implicated alone. Furthermore, adipose tissue-derived soluble factors did not favor a G0-to-G1 phase transition of MNNG-HOS cells in nonadherent oncospheres. This study indicates that adipose tissue-soluble factors activate osteosarcoma cell cycle from G1 to mitosis phases, but do not promote the transition from quiescent G0 to G1 phases. |
| **Question** | Autologous adipose tissue transfer may not be involved in the activation of dormant tumor [........] or cancer stem [........]. |
| **Options** | conditioned medium / transfer / mitosis / cell / concentrations / Adipose tissue / bone / cell cycle / MG-63 / safety / tumor / risk / injection / recurrence / metastasis / leptin / mice / Interleukin-6 / in vitro / phase transition / growth factors / serum / MNNG / needs / growth / Culture media / osteosarcoma / cell lines / HOS / lung / cancer stem cells / osteolysis / |
| **Answer** | Cells |
| **Response** | |
| **CS undergrad 1** | cell (o) |
| **CS undergrad 2** | bone (x) |
| **Bio. Grad 1** | recurrence (x) |
| **Bio. Grad 2** | cell (o) |
| **Expert 1** | cell (o) |
| **Expert 2** | cell (o) |
| **ASR model** | cell (o) |

Question 20.

| | |
|---|---|
| **Source dataset** | **BMKC_T** |
| **Context** | It is well established that natural killer (NK) cells play an important role in the immunity against cancer, while the involvement of other recently identified, NK-related innate lymphoid cells is still poorly defined. In the haploidentical hematopoietic stem cell transplantation for the therapy of high-risk leukemias, NK cells have been shown to exert a key role in killing leukemic blasts residual after conditioning. While the clinical results in the cure of leukemias are excellent, the |

| | |
|---|---|
| | exploitation of NK cells in the therapy of solid tumors is still limited and unsatisfactory. In solid tumors, NK cell function may be inhibited via different mechanisms, occurring primarily at the tumor site. The cellular interactions in the tumor microenvironment involve tumor cells, stromal cells and resident or recruited leukocytes and may favor tumor evasion from the host's defenses. In this context, a number of cytokines, growth factors and enzymes synthesized by tumor cells, stromal cells, suppressive/regulatory myeloid and lymphoid cells may substantially impair the function of different tumor-reactive effector cells, including NK cells. The identification and characterization of such mechanisms may offer clues for the development of new immunotherapeutic strategies to restore effective anti-tumor responses. In order to harness NK cell-based immunotherapies, several approaches have been proposed, including reinforcement of NK cell cytotoxicity by means of specific cytokines, antibodies or drugs. These new tools may improve NK cell function and/or increase tumor susceptibility to NK-mediated killing. Hence, the integration of NK-based immunotherapies with conventional anti-tumor therapies may increase chances of successful cancer treatment. |
| **Question** | Human natural killer cells: news in the therapy of solid [........] and high-risk leukemias. |
| **Options** | cells, stromal / role / cells / enzymes / hematopoietic stem cell transplantation / immunity / leukocytes / tumor / risk / lymphoid cells / therapies / immunotherapies / play / tumor microenvironment / identification / antibodies / cytokines / killing / growth factors / leukemias / conditioning / NK cells / drugs / reinforcement / |
| **Answer** | Tumor |
| **Response** | |
| **CS undergrad 1** | Tumor (o) |
| **CS undergrad 2** | Tumor (o) |
| **Bio. Grad 1** | Tumor (o) |
| **Bio. Grad 2** | Tumor (o) |
| **Expert 1** | Tumor (o) |
| **Expert 2** | Tumor (o) |
| **ASR model** | Tumor (o) |

Question 21.

| | |
|---|---|
| **Source dataset** | **BMKC_LS** |
| **Context** | To identify situations and thoughts that may |

| | |
|---|---|
| | precipitate or protect against loneliness experienced by patients with cancer. . Qualitative. . The hematology/oncology clinic at the Indiana University Melvin and Bren Simon Cancer Center, an outpatient oncology center in Indianapolis. . Purposive sample of 15 patients undergoing treatment for multiple myeloma or non-Hodgkin lymphoma. . Individual, semistructured qualitative interviews were conducted. Theoretical thematic analysis was used to analyze interview data.. Factors that appeared to precipitate loneliness included several situations (e.g., physical isolation, social constraints such as criticism) and thoughts (e.g., unmet expectations for visits or questions about health, belief that others do not understand their cancer experience). Several situations (e.g., social support, normal routine) and thoughts (e.g., beliefs that time alone is desirable and that others' discomfort with cancer-related discussions is normative) appeared to protect against loneliness. Certain social situations were loneliness-inducing for some patients and not for others, suggesting that patients' thoughts about their situations, rather than the situations themselves, have the greatest impact on their loneliness. . The current study fills gaps in loneliness theory by identifying cancer-related situations and thoughts that patients associate with their loneliness. Consistent with theory, patients reported feeling lonely when they had negative thoughts about their social situations. . Findings inform nursing assessment and intervention strategies to incorporate into care plans. For instance, when conducting assessments, nurses should be more attentive to patients' satisfaction with their social environment than actual characteristics of the environment. |
| **Question** | Normalizing patients' experiences and encouraging positive thoughts about others' behavior may reduce patients' [........]. |
| **Options** | environment / beliefs / nurses / cancer / social support / treatment / loneliness / multiple myeloma / satisfaction / patients / outpatient / time / University / behavior / hematology / feeling / non-Hodgkin lymphoma / isolation, social / nursing assessment / social environment / Indiana / health / interview / |
| **Answer** | Loneliness |
| **Response** | |
| **CS undergrad 1** | satisfaction (x) |
| **CS undergrad 2** | Loneliness (o) |
| **Bio. Grad 1** | Loneliness (o) |
| **Bio. Grad 2** | Loneliness (o) |
| **Expert 1** | Loneliness (o) |

| | |
|---|---|
| **Expert 2** | Loneliness (o) |
| **ASR model** | satisfaction (x) |

Question 22.

| | |
|---|---|
| **Source dataset** | **BMKC_T** |
| **Context** | Stage IV gastric cancer is lethal, and little population-based research on prognostic factors has been performed in low-incidence countries. Therefore, we investigated the consistency of the associations of patient, disease and healthcare system factors identified in previous population-based research to understand their generalizability to other low-incidence populations. A population-based, retrospective cohort study of patients diagnosed with Stage IV gastric cancer in Ontario between 1 April 2005 and 31 March 2008 was performed. Kaplan-Meier methodology and the log-rank test were used for bivariate analysis. Multivariate Cox proportional hazard regression was performed. Hazard ratios (HRs) and 95% confidence intervals (CIs) are presented. On multivariate analysis, patient, disease and healthcare system factors were independent predictors of survival. Increasing age per 10 years (HR 1.07; 95% CI 1.02-1.10), a tumor located in the gastroesophageal junction (HR 1.09; 95% CI 0.94-1.27) or middle of the stomach (HR 1.14; 95% CI 0.97-1.35), presence of carcinomatosis (HR 1.61; 95% CI 1.42-1.83) and a larger burden of metastatic disease (2-3 sites of metastatic disease: HR 1.17; 95% CI 1.03-1.32; ≥ 4 sites: HR 1.69; 95% CI 1.30-2.20) were associated with worse prognosis. Female gender, receipt of surgery, chemotherapy and radiotherapy and treatment from a high-volume, gastric cancer specialist were all associated with significantly better prognosis. In addition, there was evidence of significant geographic variation in survival. This study provides supporting evidence for patient, disease and healthcare system prognostic factors in metastatic gastric cancer. Future work investigating the role of emerging molecular and biologic information will need to take these established prognostic factors into consideration. |
| **Question** | Prognostic factors in metastatic gastric cancer: results of a population-based, retrospective cohort study in [........]. |
| **Options** | CI 1 / stomach / cohort study / gastric cancer / confidence intervals / role / associations / chemotherapy / carcinomatosis / Future / tumor / Ontario / research / gender / radiotherapy / treatment / Female / disease / patients / populations / incidence / regression / multivariate analysis / survival / prognosis / specialist / gastroesophageal junction / healthcare system / will / work / surgery / |

| | |
|---|---|
| **Answer** | Ontario |
| **Response** | |
| **CS undergrad 1** | patients (x) |
| **CS undergrad 2** | Ontario (o) |
| **Bio. Grad 1** | Ontario (o) |
| **Bio. Grad 2** | Ontario (o) |
| **Expert 1** | Ontario (o) |
| **Expert 2** | Ontario (o) |
| **ASR model** | Ontario (o) |

Question 23.

| | |
|---|---|
| **Source dataset** | **BMKC_LS** |
| **Context** | Vascular endothelial growth factor (VEGF) is highly expressed in many types of tumors, including pancreatic cancer. Tumor cellderived VEGF promotes angiogenesis and tumor progression. However, the role of VEGF in glucose metabolism remains unclear. We investigated the role and the underlying mechanism of VEGF in the glucose metabolism of pancreatic cancer cells. Pancreatic cancer cells were stimulated with VEGF165 for 1 or 2 h. The oxygen consumption rates (OCR) and extracellular acidification rates (ECAR) were measured using the Seahorse XF96 Extracellular Flux Analyzer. Glycolytic enzymes were detected by quantitative real-time PCR. Neuropilin 1 (NRP1) was silenced by shRNA in order to investigate its role in VEGF-induced glycolysis. Immunohistochemistry (IHC) was performed to identify the correlation among VEGF, NRP1 and hypoxia inducible factor 1α (HIF1α) in pancreatic cancer tissues. VEGF stimulation led to a metabolic transition from mitochondrial oxidative phosphorylation to glycolysis in pancreatic cancer. HIF1α and NRP1 protein levels were both increased after VEGF stimulation. The down-regulation of NRP1 reduced glycolysis in pancreatic cancer cells. NRP1 and VEGF levels both correlated with HIF1α expression in pancreatic tumor tissues. VEGF enhances glycolysis in pancreatic cancer via HIF1α up-regulation. |
| **Question** | NRP1 plays a key role in VEGF-induced [........]. |
| **Options** | cells / glycolysis / NRP1 / VEGF / tumor / plays / oxidative phosphorylation / Neuropilin 1 / tissues / oxygen consumption / Glycolytic enzymes / down-regulation / up-regulation / Seahorse / pancreatic cancer / Vascular endothelial growth factor / hypoxia / quantitative real-time PCR / shRNA / metabolism / Immunohistochemistry / |
| **Answer** | glucose, glycolysis |

| | |
|---|---|
| **Response** | |
| **CS undergrad 1** | glycolysis (o) |
| **CS undergrad 2** | glycolysis (o) |
| **Bio. Grad 1** | pancreatic cancer (x) |
| **Bio. Grad 2** | pancreatic cancer (x) |
| **Expert 1** | pancreatic cancer (x) |
| **Expert 2** | glycolysis (o) |
| **ASR model** | glycolysis (o) |

Question 24.

| | |
|---|---|
| **Source dataset** | **BMKC_T** |
| **Context** | Major advances have significantly improved the outcome of mantle cell lymphoma (MCL). Incorporation of rituximab to CHOP regimen, the adoption of high dose cytarabine with frontline autologous stem cell transplantation in young patients, maintenance rituximab or bortezomib based chemotherapy in elderly patients, improved the disease outcome. Bortezomib, lenalidomide, temsirolimus and ibrutinib have proven their efficacy and are approved for the use in refractory or relapsed MCL patients. Several other molecules are currently being evaluated such as cyclin dependent kinase 4/6 (CDK4/6), phosphoinositide 3-kinase (PI3K), B cell lymphoma-2 (BCL2) and Poly ADP-ribose polymerase (PARP) inhibitors. Unfortunately, we don't have specific biomarkers that could reveal which of the underlying pathways or genetic alterations are mostly involved in each individual case of MCL. Efforts should be done in this field aiming to an optimal personalized therapy. |
| **Question** | Novel targeted therapeutics for [........] - What's on the horizon? |
| **Options** | chemotherapy / mantle cell lymphoma / PI3K / cytarabine / therapy / rituximab / stem cell transplantation / disease / bortezomib / patients / temsirolimus / CHOP regimen / biomarkers / cyclin dependent kinase 4 / PARP / BCL2 / lenalidomide / B cell lymphoma / CDK4 / elderly / ibrutinib / phosphoinositide 3-kinase / adoption / MCL / Poly ADP-ribose polymerase / maintenance / |
| **Answer** | mantle cell lymphoma |
| **Response** | |
| **CS undergrad 1** | MCL (o) |
| **CS undergrad 2** | MCL (o) |
| **Bio. Grad 1** | MCL (o) |

| | |
|---|---|
| **Bio. Grad 2** | mantle cell lymphoma (o) |
| **Expert 1** | MCL (o) |
| **Expert 2** | MCL (o) |
| **ASR model** | mantle cell lymphoma (o) |

Question 25.

| | |
|---|---|
| **Source dataset** | **BMKC_LS** |
| **Context** | The resistance of cancer cells to chemotherapeutic agents represents the main problem in cancer treatment. Despite intensive research, mechanisms of resistance have not yet been fully elucidated. Six1 signaling has an important role in the expansion of progenitor cell populations during early embryogenesis. Six1 gene overexpression has been strongly associated with aggressiveness, invasiveness, and poor prognosis of different cancers. In this study, we investigated the role of Six1 signaling in resistance of MCF-7 breast cancer cells to taxanes. We first established in vitro paclitaxel-resistant MCF-7 breast cancer cells. Morphological modifications in paclitaxel-resistant cells were examined via light microscopic images and fluorescence-activated cell sorting analysis. Applying quantitative real-time polymerase chain reaction, we measured Six1, B-cell lymphoma/leukemia(BCL-2), BAX, and P53 mRNA expression levels in both non-resistant and resistant cells. Resistant cells were developed from the parent MCF-7 cells by applying increasing concentrations of paclitaxel up to 64 nM. The inhibitory concentration 50% value in resistant cells increased from 3.5 ± 0.03 to 511 ± 10.22 nM ($p = 0.015$). In paclitaxel-resistant cells, there was a significant increase in Six1 and BCL-2 mRNA levels ($p = 0.0007$) with a marked decrease in pro-apoptotic Bax mRNA expression level ($p = 0.03$); however, there was no significant change in P53 expression ($p = 0.025$). |
| **Question** | Our results suggest that identifying [........] patients with high Six1 expression and then inhibition of Six1 signaling can improve the efficiency of chemotherapeutic agents in the induction of apoptosis. |
| **Options** | breast cancer / role / Six1 / cells / taxanes / concentrations / MCF-7 / inhibitory concentration 50 / embryogenesis / cancer / research / treatment / inhibition / paclitaxel / populations / patients / progenitor cell / prognosis / in vitro / P53 / BCL-2 / parent / BAX / B-cell lymphoma / leukemia / efficiency / MCF-7 cells / quantitative real-time polymerase chain reaction / fluorescence-activated cell sorting / light / apoptosis / mRNA / |
| **Answer** | Cancer |
| **Response** | |

| | |
|---|---|
| **CS undergrad 1** | paclitaxel (x) |
| **CS undergrad 2** | concentrations (x) |
| **Bio. Grad 1** | breast cancer (x) |
| **Bio. Grad 2** | breast cancer (x) |
| **Expert 1** | breast cancer (x) |
| **Expert 2** | breast cancer (x) |
| **ASR model** | breast cancer (x) |

Question 26.

| | |
|---|---|
| **Source dataset** | **BMKC_T** |
| **Context** | Uterine leiomyomatosis and especially submucosal myomas hamper the outcomes of Assisted Reproductive Techniques (ART). Even though surgical treatment eliminates gross anatomical anomalies, medical treatment should be encouraged to improve the overall structure of the uterus, thereby enabling ART. We report the case of an infertile female patient suffering from symptomatic uterine fibromatosis, who received 5 mg/day ulipristal acetate (UPA), a selective progesterone receptor modulator (SPRMs), for three months before and after hysteroscopic myomectomy. Uterine bleeding reduced on the eight days of treatment, with a subsequent improvement of pelvic pain. Under transvaginal ultrasound the uterus appeared globally enlarged with a diffuse leiomyomatosis of the myometrial layer. Saline infusion showed a markedly distorted cavity due two submucosal myomas (sized 31 × 24 mm and 21 × 19 mm, respectively) and one intramural myoma (37 × 34 mm). After three months the size of the myomas was reduced by 30-40%, allowing the hysteroscopic removal of the submucosal fibroids and the bigger intramural one. The smaller fibroids involving the myometrial layer were instead too diffused to be removed. At the conclusion of the subsequent cycle of UPA, the overall appearance of the cavity had improved, and the endometrial layer was regular, allowing the patient to undergo in vitro fertilization (IVF). There was no adverse effect related to treatment, and the endometrial biopsy did not reveal any histologic change. UPA seems to have a triple effect: it ensures prompt symptom relief, it reduces the size of the myomas enabling surgery and it improves the morphology of the uterus. |
| **Question** | Ulipristal acetate prior to [........] in a female patient affected by uterine fibroids: a case report. |
| **Options** | biopsy / fibroids / in vitro fertilization / report / who / treatment / female / patient / suffering / pelvic pain / overall / ART / IVF / |

| | |
|---|---|
| | ulipristal acetate / UPA / myomectomy. Uterine / leiomyomatosis / Uterine / Assisted Reproductive Techniques / myoma / fibromatosis / progesterone receptor / surgery / uterus /<br>in vitro fertilization |
| **Answer** | in vitro fertilization |
| **Response** | |
| **CS undergrad 1** | fibromatosis (x) |
| **CS undergrad 2** | uterus (x) |
| **io. Grad 1** | UPA (x) |
| **Bio. Grad 2** | treatment (x) |
| **Expert 1** | surgery (x) |
| **Expert 2** | ART (x) |
| **ASR model** | in vitro fertilization (o) |

Question 27.

| | |
|---|---|
| **Source dataset** | **BMKC_LS** |
| **Context** | This is an update of the original Cochrane review published in Issue 4, 2000. Intestinal obstruction commonly occurs in progressive advanced gynaecological and gastrointestinal cancers. Management of these patients is difficult due to the patients' deteriorating mobility and function (performance status), the lack of further chemotherapeutic options, and the high mortality and morbidity associated with palliative surgery. There are marked variations in clinical practice concerning surgery in these patients between different countries, gynaecological oncology units and general hospitals, as well as referral patterns from oncologists under whom these patients are often admitted. To assess the efficacy of surgery for intestinal obstruction due to advanced gynaecological and gastrointestinal cancer. We searched the following databases for the original review in 2000 and again for this update in June 2015: CENTRAL (2015, Issue 6); MEDLINE (OVID June week 1 2015); and EMBASE (OVID week 24, 2015).We also searched relevant journals, bibliographic databases, conference proceedings, reference lists, grey literature and the world wide web for the original review in 2000; we also used personal contact. This searching of other resources yielded very few additional studies. The Cochrane Pain, Palliative and Supportive Care Review Group no longer routinely handsearch journals. For these reasons, we did not repeat the searching of other resources for the June 2015 update. As the review concentrates on the 'best evidence' |

| | |
|---|---|
| | available for the role of surgery in malignant bowel obstruction in known advanced gynaecological and gastrointestinal cancer we kept the inclusion criteria broad (including both prospective and retrospective studies) so as to include all studies relevant to the question. We sought published trials reporting on the effects of surgery for resolving symptoms in malignant bowel obstruction for adult patients with known advanced gynaecological and gastrointestinal cancer. We used data extraction forms to collect data from the studies included in the review. Two review authors extracted the data independently to reduce error. Owing to concerns about the risk of bias we decided not to conduct a meta-analysis of data and we have presented a narrative description of the study results. We planned to resolve disagreements by discussion with the third review author. In total we have identified 43 studies examining 4265 participants. The original review included 938 patients from 25 studies. The updated search identified an additional 18 studies with a combined total of 3327 participants between 1997 and June 2015. The results of these studies did not change the conclusions of the original review. No firm conclusions can be drawn from the many retrospective case series so the role of surgery in malignant bowel obstruction remains controversial. Clinical resolution varies from 26.7% to over 68%, though it is often unclear how this is defined. Despite being an inadequate proxy for symptom resolution or quality of life, the ability to feed orally was a popular outcome measure, with success rates ranging from 30% to 100%. Rates of re-obstruction varied, ranging from 0% to 63%, though time to re-obstruction was often not included. Postoperative morbidity and mortality also varied widely, although again the definition of both of these surgical outcomes differed between many of the papers. There were no data available for quality of life. The reporting of adverse effects was variable and this has been described where available. Where discussed, surgical procedures varied considerably and outcomes were not reported by specific intervention. Using the 'Risk of bias' assessment tool, most included studies were at high risk of bias for most domains. The role of surgery in malignant bowel obstruction needs careful evaluation, using validated outcome measures of symptom control and quality of life scores. Further information could include re-obstruction rates together with the morbidity associated with the various surgical procedures. Currently, bowel obstruction is managed empirically and there are marked variations in clinical practice by different units. |
| **Question** | In order to compare outcomes in malignant bowel obstruction, there needs to be a greater degree of standardisation of management. Since the last version of this [........] none of the new |

| | |
|---|---|
| | included studies have provided additional information to change the conclusions. |
| **Options** | role / databases / world wide web / evaluation / Risk / mortality / resources / papers / patients / retrospective studies / meta-analysis / literature / palliative surgery / bibliographic databases / Pain / time / adult / outcome measure / ability / general hospitals / intestinal obstruction / needs / forms / morbidity / gastrointestinal cancer / quality of life / bias / proxy / review / surgery / referral / MEDLINE / |
| **Answer** | review |
| **Response** | |
| **CS undergrad 1** | review (o) |
| **CS undergrad 2** | evaluation (x) |
| **Bio. Grad 1** | review (o) |
| **Bio. Grad 2** | review (o) |
| **Expert 1** | review (o) |
| **Expert 2** | review (o) |
| **ASR model** | review (o) |

Question 28.

| | |
|---|---|
| **Source dataset** | **BMKC_T** |
| **Context** | Primary sclerosing cholangitis (PSC) is a chronic progressive disease, usually associated with underlying inflammatory bowel diseases (IBDs), with a prevalence of 60-80% in western countries. Herein, we review the current knowledge about the association between PSC and IBD in terms of clinical approach and long-term patient management. A PubMed search was conducted for English-language publications from 2000 through 2015 using the following keywords: primary sclerosing cholangitis, inflammatory bowel disease, ulcerative colitis, Crohn's disease, diagnosis, therapy, follow-up, and epidemiology. In terms of diagnosis, liver function tests and histology are currently used. The medical treatment options for PSC associated with IBD do not differ from the cases of PSC alone, and include ursodeoxycholic acid and immunosuppressive agents. These treatments do not seem to improve survival, even if ursodeoxycholic acid given at low doses may be chemopreventive against colorectal cancer (CRC). Liver transplantation is the only potential curative therapy for PSC with reported survival rates of 85 and 70% at 5 and 10 years after transplant; however, there is a risk for PSC recurrence, worsening of IBD activity, and de-novo IBD occurrence after liver transplantation. PSC-IBD represents an |

| | |
|---|---|
| | important public health concern, especially in view of the increased risk for malignancy, including CRC. Long-life annual surveillance colonoscopy is usually recommended, although the exact timescale is still unclear. Further studies are required both to clarify whether annual colonoscopy is cost-effective, especially in younger patients, and to identify potential pharmaceutical agents and genetic targets that may retard disease progression and protect against CRC. |
| **Question** | Primary sclerosing cholangitis associated with inflammatory bowel [........]: an update. |
| **Options** | |
| **Answer** | disease |
| **Response** | |
| **CS undergrad 1** | disease (o) |
| **CS undergrad 2** | disease (o) |
| **Bio. Grad 1** | disease (o) |
| **Bio. Grad 2** | disease (o) |
| **Expert 1** | disease (o) |
| **Expert 2** | disease (o) |
| **ASR model** | disease (o) |

Question 29.

| | |
|---|---|
| **Source dataset** | **BMKC_LS** |
| **Context** | Peru has high cervical cancer incidence and mortality rates compared to other Andean countries. Therefore, partnerships between governmental and international organizations have targeted rural areas of Peru to receive cervical cancer screening via outreach campaigns. Previous studies have found a relationship between a person's social networks and cancer screening behaviors. Screening outreach campaigns conducted by the nonprofit organization CerviCusco created an opportunity for a social network study to examine cervical cancer screening history and social network characteristics in a rural indigenous community that participated in these campaigns in 2012 and 2013. The aim of this study was to explore social network characteristics in this community related to receipt of cervical cancer screening following the campaigns. An egocentric social network questionnaire was used to collect cross-sectional network data on community participants. Each survey participant (ego) was asked to name six other women they knew (alters) and identify the nature of their relationship or tie (family, friend, neighbor, other), residential closeness (within 5 km), length of |

| | |
|---|---|
| | time known, frequency of communication, topics of conversation, and whether they lent money to the person, provided childcare or helped with transportation. In addition, each participant was asked to report the nature of the relationship between all alters identified (e.g., friend, family, or neighbor). Bivariate and multivariate analyses were used to explore the relationship between Pap test receipt at the CerviCusco outreach screening campaigns and social network characteristics. Bivariate results found significant differences in percentage of alter composition for neighbors and family, and for mean number of years known, mean density, and mean degree centrality between women who had received a Pap test (n = 19) compared to those who had not (n = 50) (p's < 0.05). The final logistic regression model was statistically significant ($\chi 2$ (2) = 20.911, p < .001). The model included the variables for percentage of family alter composition and mean density, and it explained 37.8 % (Nagelkerke R (2)) of the variance in Pap test receipt, correctly classifying 78.3 % of cases. Those women with higher percentages of family alter composition and higher mean density in their ego networks were less likely to have received a Pap test at the CerviCusco campaigns. According to this exploratory study, female neighbors more than family members may have provided an important source of social support for healthcare related decisions related to receipt of a Pap test. |
| **Question** | Future studies should collect longitudinal [........] data on participants to measure the network effects of screening interventions in rural indigenous communities in Latin American countries experiencing the highest burden of cervical cancer. |
| **Options** | transportation / friend / ego / Future / social network / report / mortality rates / communication / who / Peru / female / incidence / multivariate analyses / survey / time / nature / behaviors / nonprofit organization / organizations / Pap test / cervical cancer / history / healthcare / screening / cancer screening / name / community / family / person / measure / logistic regression / women / |
| **Answer** | social network |
| **Response** | |
| **CS undergrad 1** | social network (o) |
| **CS undergrad 2** | social network (o) |
| **Bio. Grad 1** | cervical cancer (x) |
| **Bio. Grad 2** | social network (o) |
| **Expert 1** | survey (x) |
| **Expert 2** | social network (o) |

| | |
|---|---|
| **ASR model** | social network (o) |

Question 30.

| | |
|---|---|
| **Source dataset** | **BMKC_T** |
| **Context** | Irinotecan is approved and widely administered to metastatic colorectal cancer (mCRC) patients; however, it can cause severe toxicities including neutropenia and diarrhea. The polymorphisms of genes encoding drug-metabolizing enzymes can play a crucial role in the increased susceptibility of cancer patients to chemotherapy toxicity. Therefore, we plan to explore the effect of the genetic polymorphism of uridine diphosphate glucuronosyltransferase 1A1 (UGT1A1) for irinotecan detoxification in mCRC patients. This trial will compare the clinical outcomes and side effects observed in mCRC patients treated with bevacizumab plus 5-fluorouracil/leucovorin/irinotecan (FOLFIRI) with and without UGT1A1 genotyping and irinotecan dose escalation. A total of 400 mCRC patients were randomized into a study group and a control group. This trial is a prospective, multicenter, randomized clinical trial comparing UGT1A1 promoter polymorphism for irinotecan dose escalation in mCRC patients administered with bevacizumab plus FOLFIRI as the first-line setting. The enrolled patients were randomly assigned to one of two groups, a study group and a control group, on the basis of receiving UGT1A1 genotyping or not. The study group receive a biweekly FOLFIRI regimen, with irinotecan dose escalation based on UGT1A1 genotyping; whereas the control group receive the conventional biweekly FOLFIRI regimen without UGT1A1 genotyping. The clinicopathological features, response rates, toxicity, and progression-free survival or overall survival will be compared between the two groups. Patients with mCRC undergoing UGT1A1 genotyping may receive escalated doses of irinotecan for a potentially more favorable clinical response and outcome, in addition to comparable toxicities. Such personalized medicine based on genotyping may be feasible for clinical practice. NCT02256800 . Date of registration: 3 October 2014. Date of first patient randomized: 16 January 2015. |
| **Question** | Prospective analysis of UGT1A1 promoter polymorphism for irinotecan dose escalation in metastatic colorectal [........] patients treated with bevacizumab plus FOLFIRI as the first-line setting: study protocol for a randomized controlled trial. |
| **Options** | FOLFIRI / leucovorin / diarrhea / role / chemotherapy / enzymes / UGT1A1 / control group / neutropenia / 1A1 / cancer / play / patients / colorectal cancer / genetic polymorphism / survival / |

| | |
|---|---|
| | overall / clinical trial / progression-free survival / personalized medicine / genes / bevacizumab / 5-fluorouracil / will / glucuronosyltransferase / uridine diphosphate / irinotecan / |
| **Answer** | cancer |
| **Response** | |
| **CS undergrad 1** | cancer (o) |
| **CS undergrad 2** | cancer (o) |
| **Bio. Grad 1** | cancer (o) |
| **Bio. Grad 2** | cancer (o) |
| **Expert 1** | cancer (o) |
| **Expert 2** | cancer (o) |
| **ASR model** | cancer (o) |

Question 31.

| **Source dataset** | **BMKC_LS** |
|---|---|
| **Context** | The Children's Oncology Group (COG) publishes consensus guidelines with screening recommendations for early identification of treatment-related morbidities among childhood cancer survivors. We sought to estimate the yield of recommended yearly urinalysis screening for genitourinary complications as per Version 3.0 of the COG Long-Term Follow-Up Guidelines and identify possible risk factors for abnormal screening in a survivor population. A database of pediatric cancer survivors evaluated between January 2008 and March 2012 at Children's Healthcare of Atlanta was queried for survivors at risk for genitourinary late effects. The frequency of abnormal urinalyses (protein ≥1+ and/or presence of glucose and/or ≥5 red blood cells per high power field) was estimated. Risk factors associated with abnormal screening were identified. Chart review identified 773 survivors (57% male; 67% Caucasian; 60% leukemia/lymphoma survivors; mean age at diagnosis, 5.7 years [range: birth to 17.7 years]; time from diagnosis to initial screening, 7.6 years [range: 2.3 to 21.5 years]) who underwent urinalysis. Abnormal results were found in 78 (5.3%) of 1,484 total urinalyses. Multivariable analysis revealed higher dose ifosfamide (odds ratio [OR] = 6.8, 95% confidence interval [CI] 2.9-16.0) and total body irradiation (TBI, OR = 3.0, 95% CI 1.0-8.4) as significant risk factors for abnormal initial urinalysis screening. Pediatric cancer survivors exposed to higher dose ifosfamide or TBI may be at higher risk of abnormal findings on urinalysis screening. |
| **Question** | Targeted screening of these higher [........] patients should be |

| | |
|---|---|
| | considered. |
| **Options** | CI 1 / glucose / red blood cells / confidence interval / database / urinalysis / ifosfamide / cancer / risk / risk factors / who / treatment / male / population / patients / identification / time / guidelines / diagnosis / Children / leukemia / Healthcare / screening / morbidities / birth / power / total body irradiation / odds ratio / survivors / review / consensus / |
| **Answer** | risk |
| **Response** | |
| **CS undergrad 1** | ifosfamide (x) |
| **CS undergrad 2** | red blood cells (x) |
| **Bio. Grad 1** | leukemia (x) |
| **Bio. Grad 2** | ifosfamide (x) |
| **Expert 1** | urinalysis (x) |
| **Expert 2** | treatment (x) |
| **ASR model** | Risk (o) |

Question 32.

| | |
|---|---|
| **Source dataset** | **BMKC_T** |
| **Context** | Widespread disparities in care have been documented in women with gynecologic cancer in the United States. This study was designed to determine whether structural barriers to optimal care were present during the preoperative period for patients with gynecologic cancer. A retrospective review was conducted for patients undergoing surgery for a gynecologic malignancy at a public hospital or a private hospital staffed by the same team of gynecologic oncologists between July 1, 2013 and July 1, 2014. Two hundred fifty-seven cases were included for analysis (public hospital, 69; private hospital, 188). Patients treated at the private hospital were older (58 vs 52 years; P = .004) and had similar medical comorbidities (median Charlson comorbidity index at both hospitals, 6) but required fewer hospital visits in preparation for surgery (2 vs 4; P < .001). Public hospital patients had a longer wait time from the diagnosis of disease to surgery (63 vs 34 days; P < .001). According to a multiple linear regression model, the public hospital setting was associated with a longer interval from diagnosis to surgery with adjustments for the insurance status, age at diagnosis, cancer stage, and number of preoperative hospital visits (P < .001). Patients at the public hospital were subject to a greater number of preoperative visits and had to wait longer for surgery than patients at the private |

| | |
|---|---|
| | hospital. Attempts to reduce health care disparities should focus on improving efficiency in health care delivery systems once contact has been established. Cancer 2016;122:859-67. © 2016 American Cancer Society. |
| **Question** | Preoperative experience for public hospital [........] with gynecologic cancer: Do structural barriers widen the gap? |
| **Options** | United States / public hospital / adjustments / American Cancer Society / cancer / disease / preoperative period / Patients / private hospital / insurance status / health care disparities / comorbidities / time / diagnosis / efficiency / health care delivery / hospital / linear regression / review / surgery / women / |
| **Answer** | patients |
| **Response** | |
| **CS undergrad 1** | health care delivery (x) |
| **CS undergrad 2** | surgery (x) |
| **Bio. Grad 1** | disease (x) |
| **Bio. Grad 2** | surgery (x) |
| **Expert 1** | Patients (o) |
| **Expert 2** | health care disparities (x) |
| **ASR model** | Patients (o) |

Question 33.

| **Source dataset** | **BMKC_LS** |
|---|---|
| **Context** | Targeting the TGF-β1 pathway for breast cancer metastasis therapy has become an attractive strategy. We have previously demonstrated that naringenin significantly reduced TGF-β1 levels in bleomycin-induced lung fibrosis and effectively prevented pulmonary metastases of tumors. This raised the question of whether naringenin can block TGF-β1 secretion from breast cancer cells and inhibit their pulmonary metastasis. We transduced a lentiviral vector encoding the mouse Tgf-β1 gene into mouse breast carcinoma (4T1-Luc2) cells and inoculated the transformant cells (4T1/TGF-β1) into the fourth primary fat pat of Balb/c mice. Pulmonary metastases derived from the primary tumors were monitored using bioluminescent imaging. Spleens, lungs and serum (n = 18-20 per treatment group) were analyzed for immune cell activity and TGF-β1 level. The mechanism whereby naringenin decreases TGF-β1 secretion from breast cancer cells was investigated at different levels, including Tgf-β1 transcription, mRNA stability, translation, and extracellular release. In contrast to the null-vector control (4T1/RFP) tumors, |

| | |
|---|---|
| | extensive pulmonary metastases derived from 4T1/TGF-β1 tumors were observed. Administration of the TGF-β1 blocking antibody 1D11 or naringenin showed an inhibition of pulmonary metastasis for both 4T1/TGF-β1 tumors and 4T1/RFP tumors, resulting in increased survival of the mice. Compared with 4T1/RFP bearing mice, systemic immunosuppression in 4T1/TGF-β1 bearing mice was observed, represented by a higher proportion of regulatory T cells and myeloid-derived suppressor cells and a lower proportion of activated T cells and INFγ expression in CD8(+) T cells. These metrics were improved by administration of 1D11 or naringenin. However, compared with 1D11, which neutralized secreted TGF-β1 but did not affect intracellular TGF-β1 levels, naringenin reduced the secretion of TGF-β1 from the cells, leading to an accumulation of intracellular TGF-β1. Further experiments revealed that naringenin had no effect on Tgf-β1 transcription, mRNA decay or protein translation, but prevented TGF-β1 transport from the trans-Golgi network by inhibiting PKC activity. Naringenin blocks TGF-β1 trafficking from the trans-Golgi network by suppressing PKC activity, resulting in a reduction of TGF-β1 secretion from breast cancer cells. |
| **Question** | This finding suggests that naringenin may be an attractive therapeutic candidate for [........]-β1 related diseases. |
| **Options** | affect / CD8 / breast cancer / cells / bleomycin / Tgf / tumors / therapy / inhibition / metrics / PKC / immunosuppression / diseases / metastases / mice / naringenin / Spleens / survival / mRNA stability / protein translation / Balb/c mice / serum / administration / gene / trans-Golgi network / fibrosis / secretion / lungs / regulatory T cells / T cells / RFP / |
| **Answer** | TGF |
| **Response** | |
| **CS undergrad 1** | Tgf (o) |
| **CS undergrad 2** | Tgf (o) |
| **Bio. Grad 1** | Tgf (o) |
| **Bio. Grad 2** | Tgf (o) |
| **Expert 1** | breast cancer (x) |
| **Expert 2** | TGF (o) |
| **ASR model** | Metastasis (x) |

Question 34.

| | |
|---|---|
| **Source dataset** | **BMKC_T** |
| **Context** | The Reproducibility Project: Cancer Biology seeks to address |

| | |
|---|---|
| | growing concerns about reproducibility in scientific research by conducting replications of selected experiments from a number of high-profile papers in the field of cancer biology. The papers, which were published between 2010 and 2012, were selected on the basis of citations and Altmetric scores (Errington et al., 2014). This Registered Report describes the proposed replication plan of key experiments from 'Fusobacterium nucleatum infection is prevalent in human colorectal carcinoma' by Castellarin and colleagues published in Genome Research in 2012 (Castellarin et al., 2012). The experiment to be replicated is reported in Figure 2. Here, Castellarin and colleagues performed a metagenomic analysis of colorectal carcinoma (CRC) to identify potential associations between inflammatory microorganisms and gastrointestinal cancers. They conducted quantitative real-time PCR on genomic DNA isolated from tumor and matched normal biopsies from a patient cohort and found that the overall abundance of Fusobacterium was 415 times greater in CRC versus adjacent normal tissue. These results confirmed earlier studies and provide evidence for a link between tissue-associated bacteria and tumorigenesis. The Reproducibility Project: Cancer Biology is a collaboration between the Center for Open Science and Science Exchange and the results of the replications will be published in eLife. |
| **Question** | Registered report: [........] nucleatum infection is prevalent in human colorectal carcinoma. |
| **Options** | biopsies / associations / tumor / Report / research / metagenomic / papers / patient / tissue / colorectal carcinoma / Fusobacterium nucleatum / Science / times / overall / bacteria / human / biology / tumorigenesis / infection / collaboration / quantitative real-time PCR / DNA / gastrointestinal cancers / will / Fusobacterium / Genome / |
| **Answer** | Fusobacterium |
| **Response** | |
| **CS undergrad 1** | Fusobacterium (o) |
| **CS undergrad 2** | Fusobacterium (o) |
| **Bio. Grad 1** | Fusobacterium (o) |
| **Bio. Grad 2** | Fusobacterium (o) |
| **Expert 1** | Fusobacterium (o) |
| **Expert 2** | Fusobacterium (o) |
| **ASR model** | Fusobacterium (o) |

Question 35.

| | |
|---|---|
| **Source** | **BMKC_LS** |

| | |
|---|---|
| **dataset** | |
| **Context** | In this study, we investigated the antitumor activity of icariin (ICA) in human esophageal squamous cell carcinoma (ESCC) in vitro and in vivo and explored the role of endoplasmic reticulum stress (ERS) signaling in this activity. ICA treatment resulted in a dose- and time-dependent decrease in the viability of human EC109 and TE1 ESCCs. Additionally, ICA exhibited strong antitumor activity, as evidenced by reductions in cell migration, adhesion, and intracellular glutathione (GSH) levels and by increases in the EC109 and TE1 cell apoptotic index, Caspase 9 activity, reactive oxygen species (ROS) level, and nicotinamide adenine dinucleotide phosphate (NADPH) oxidase activity. Furthermore, ICA treatments upregulated the levels of ERS-related molecules (p-PERK, GRP78, ATF4, p-eIF2α, and CHOP) and a pro-apoptotic protein (PUMA) and simultaneously downregulated an anti-apoptotic protein (Bcl2) in the two ESCC cell lines. The downregulation of ERS signaling using eIF2α siRNA desensitized EC109 and TE1 cells to ICA treatment, and the upregulation of ERS signaling using thapsigargin sensitized EC109 and TE1 cells to ICA treatment. |
| **Question** | In summary, ERS activation may represent a mechanism of action for the anticancer activity of ICA in ESCCs, and the activation of ERS signaling may represent a novel therapeutic intervention for [........] esophageal cancer. |
| **Options** | icariin / esophageal cancer / NADPH / role / cells / ICA / nicotinamide adenine dinucleotide phosphate / squamous cell carcinoma / therapeutic / Caspase 9 / TE1 / oxidase / downregulation / upregulation / GRP78 / time / CHOP / in vitro / Bcl2 / reactive oxygen species / thapsigargin / ATF4 / human / glutathione / EC109 / ROS / PUMA / GSH / PERK / cell migration / endoplasmic reticulum stress / cell lines / siRNA / |
| **Answer** | human |
| **Response** | |
| **CS undergrad 1** | human (o) |
| **CS undergrad 2** | cell lines (x) |
| **Bio. Grad 1** | human (o) |
| **Bio. Grad 2** | human (o) |
| **Expert 1** | human (o) |
| **Expert 2** | squamous cell carcinoma (x) |
| **ASR model** | human (o) |

Question 36.

| | |
|---|---|
| **Source** | **BMKC_T** |

| | |
|---|---|
| **dataset** | |
| **Context** | Epstein-Barr virus (EBV) is an oncogenic herpesvirus that has been causally linked to the development of B-cell and epithelial malignancies. Early after infection, EBV induces a transient period of hyperproliferation that is suppressed by the activation of the DNA damage response and a G1/S-phase growth arrest. This growth arrest prevents long-term outgrowth of the majority of infected cells. We developed a method to isolate and characterize infected cells that arrest after this early burst of proliferation and integrated gene expression and metabolic profiling to gain a better understanding of the pathways that attenuate immortalization. We found that the arrested cells have a reduced level of mitochondrial respiration and a decrease in the expression of genes involved in the TCA cycle and oxidative phosphorylation. Indeed, the growth arrest in early infected cells could be rescued by supplementing the TCA cycle. Arrested cells were characterized by an increase in the expression of p53 pathway gene targets, including sestrins leading to activation of AMPK, a reduction in mTOR signaling, and, consequently, elevated autophagy that was important for cell survival. Autophagy was also critical to maintain early hyperproliferation during metabolic stress. Finally, in assessing the metabolic changes from early infection to long-term outgrowth, we found concomitant increases in glucose import and surface glucose transporter 1 (GLUT1) levels, leading to elevated glycolysis, oxidative phosphorylation, and suppression of basal autophagy. Our study demonstrates that oncogene-induced senescence triggered by a combination of metabolic and genotoxic stress acts as an intrinsic barrier to EBV-mediated transformation. |
| **Question** | Metabolic stress is a barrier to Epstein-Barr virus-mediated [........] immortalization. |
| **Options** | mTOR / glucose / gene expression / EBV / Autophagy / cells / senescence / glycolysis / B-cell / cell survival / oxidative phosphorylation / metabolic stress / TCA / transient / p53 / GLUT1 / genotoxic stress / genes / oncogene / infection / growth / method / respiration / understanding / AMPK / glucose transporter / |
| **Answer** | B-cell |
| **Response** | |
| **CS undergrad 1** | transient (x) |
| **CS undergrad 2** | understanding (x) |
| **Bio. Grad 1** | B-cell (o) |
| **Bio. Grad 2** | cells (x) |

| | |
|---|---|
| **Expert 1** | cells (x) |
| **Expert 2** | senescence (x) |
| **ASR model** | B-cell (o) |

Question 37.

| | |
|---|---|
| **Source dataset** | **BMKC_LS** |
| **Context** | Oral cancer is the sixth most common cause of death from cancer with an estimated 400,000 deaths worldwide and a low (50%) 5-year survival rate. The most common form of oral cancer is oral squamous cell carcinoma (OSCC). OSCC is highly inflammatory and invasive, and the degree of inflammation correlates with tumor aggressiveness. The G protein-coupled receptor protease-activated receptor-2 (PAR-2) plays a key role in inflammation. PAR-2 is activated via proteolytic cleavage by trypsin-like serine proteases, including kallikrein-5 (KLK5), or by treatment with activating peptides. PAR-2 activation induces G protein-α-mediated signaling, mobilizing intracellular calcium and Nf-κB signaling, leading to the increased expression of pro-inflammatory mRNAs. Little is known, however, about PAR-2 regulation of inflammation-related microRNAs. Here, we assess PAR-2 expression and function in OSCC cell lines and tissues. Stimulation of PAR-2 activates Nf-κB signaling, resulting in RelA nuclear translocation and enhanced expression of pro-inflammatory mRNAs. Concomitantly, suppression of the anti-inflammatory tumor suppressor microRNAs let-7d, miR-23b, and miR-200c was observed following PAR-2 stimulation. Analysis of orthotopic oral tumors generated by cells with reduced KLK5 expression showed smaller, less aggressive lesions with reduced inflammatory infiltrate relative to tumors generated by KLK5-expressing control cells. |
| **Question** | Together, these data support a model wherein KLK5-mediated [........] activation regulates the expression of inflammation-associated mRNAs and microRNAs, thereby modulating progression of oral tumors. |
| **Options** | role / let-7d / deaths / cells / inflammation / cancer / squamous cell carcinoma / treatment / regulation / plays / serine proteases / microRNAs / Oral cancer / tissues / survival rate / protease-activated receptor-2 / miR-200c / cause of death / PAR-2 / KLK5 / cell lines / G protein-coupled receptor / G protein / miR-23b / mRNAs / peptides / trypsin / |
| **Answer** | PAR-2 |
| **Response** | |
| **CS undergrad 1** | PAR-2 (o) |

| | |
|---|---|
| **CS undergrad 2** | peptides (x) |
| **Bio. Grad 1** | PAR-2 (o) |
| **Bio. Grad 2** | PAR-2 (o) |
| **Expert 1** | PAR-2 (o) |
| **Expert 2** | inflammation (x) |
| **ASR model** | PAR-2 (o) |

Question 38.

| | |
|---|---|
| **Source dataset** | **BMKC_T** |
| **Context** | Prostate cancer is the second most frequently diagnosed cancer in males worldwide and is commonly associated with metastasis. Moreover, in prostate cancer, aminopeptidase N (APN) expression is closely correlated with metastasis. Ubenimex, an APN inhibitor, is widely used as an adjunct therapy for cancer, enhancing the function of immunocompetent cells and conferring antitumor effects. However, due to the low expression of APN, it is rarely used to treat prostate cancer. Recently, the induction of autophagy as a molecular mechanism has been strongly connected with tumor cell death. Thus, we investigated whether ubenimex could inhibit cell proliferation, migration and invasion by downregulating APN expression to induce autophagic cell death in prostate cancer cells. The LNCaP and PC-3 cell lines were treated with different doses of ubenimex. Cell viability was measured using growth curve analysis and WST-8 proliferation assay. Autophagic cell death was assessed using fluorescence microscopy and acridine orange/ethidium bromide (AO/EB) staining. Protein expression was assessed by immunofluorescence and western blot analyses. Autophagosomes were evaluated using transmission electron microscopy. Wound-healing migration assays were performed to determine the migratory ability of the PC-3 cells. In addition, nude mice were used in the present study to examine PC-3 cell proliferation in vivo. The results revealed that APN expression differed between the metastatic and non-metastatic prostate cancer cells. In addition, ubenimex inhibited APN expression in the prostate cancer cells. Ubenimex increased prostate cancer cell death, as determined using the lactate dehydrogenase (LDH) cytotoxicity assay. This effect was accompanied by increased levels of LC3B. Furthermore, ubenimex inhibited PC-3 cell proliferation in vivo and in vitro. Ubenimex inhibited the cell migration and invasion in prostate cancer cells by downregulating APN expression. Finally, ubenimex induced autophagic cell death in both metastatic and non-metastatic |

| | |
|---|---|
| | prostate cancer cells. Based on these results, ubenimex appears to be an excellent adjunctive therapy for the treatment of prostate cancer. |
| **Question** | Ubenimex inhibits cell proliferation, migration and invasion by inhibiting the expression of APN and inducing autophagic cell death in prostate cancer [........]. |
| **Options** | cell proliferation / LNCaP / nude mice / staining / western blot / cells / death / autophagic cell death / LC3B / fluorescence microscopy / APN / tumor / Cell viability / acridine orange/ethidium bromide / Wound-healing / treatment / males / metastasis / Ubenimex / prostate cancer / WST-8 / aminopeptidase N / cell death / in vitro / transmission electron microscopy / ability / immunofluorescence / growth / cell migration / cell lines / lactate dehydrogenase / |
| **Answer** | cells |
| **Response** | |
| **CS undergrad 1** | cells (o) |
| **CS undergrad 2** | cells (o) |
| **Bio. Grad 1** | metastasis (x) |
| **Bio. Grad 2** | cells (o) |
| **Expert 1** | cells (o) |
| **Expert 2** | cell lines (x) |
| **ASR model** | cells (o) |

Question 39.

| | |
|---|---|
| **Source dataset** | **BMKC_LS** |
| **Context** | Human colon cancers commonly harbor loss of function mutations in APC, a repressor of the canonical WNT pathway, thus leading to hyperactive WNT-TCF signaling. Re-establishment of Apc function in mice, engineered to conditionally repress Apc through RNAi, resolve the intestinal tumors formed due to hyperactivated Wnt-Tcf signaling. These and other results have prompted the search for specific WNT pathway antagonists as therapeutics for clinically problematic human colon cancers and associated metastases, which remain largely incurable. This widely accepted view seems at odds with a number of findings using patient-derived material: Canonical TCF targets are repressed, instead of being hyperactivated, in advanced colon cancers, and repression of TCF function does not generally result in tumor regression in xenografts. The results of a number of genetic mouse studies have also suggested that canonical WNT-TCF signaling drives metastases, but direct in vivo tests are |

| | |
|---|---|
| | lacking, and, surprisingly, TCF repression can enhance directly seeded metastatic growth. Here we have addressed the abilities of enhanced and blocked WNT-TCF signaling to alter tumor growth and distant metastases using xenografts of advanced human colon cancers in mice. We find that endogenous WNT-TCF signaling is mostly anti-metastatic since downregulation of TCF function with dnTCF generally enhances metastatic spread. Consistently, elevating the level of WNT signaling, by increasing the levels of WNT ligands, is not generally pro-metastatic. Our present and previous data reveal a heterogeneous response to modulating WNT-TCF signaling in human cancer cells. Nevertheless, the findings that a fraction of colon cancers tested require WNT-TCF signaling for tumor growth but all respond to repressed signaling by increasing metastases beg for a reevaluation of the goal of blocking WNT-TCF signaling to universally treat colon cancers. |
| **Question** | Our data suggest that WNT-[........] blockade may be effective in inhibiting tumor growth in only a subset of cases but will generally boost metastases. |
| **Options** | cells / tumor / therapeutics / metastases / patient / mice / APC / regression / downregulation / xenografts / repression / human / drives / abilities / TCF / growth / WNT pathway / RNAi / will / ligands / colon cancers / goal / mutations / |
| **Answer** | TCF |
| **Response** | |
| **CS undergrad 1** | TCF (o) |
| **CS undergrad 2** | TCF (o) |
| **Bio. Grad 1** | TCF (o) |
| **Bio. Grad 2** | TCF (o) |
| **Expert 1** | TCF (o) |
| **Expert 2** | TCF (o) |
| **ASR model** | TCF (o) |

Question 40.

| | |
|---|---|
| **Source dataset** | **BMKC_T** |
| **Context** | To assess sex hormones, leptin and insulin-resistance in men with prostate cancer (PCa) and benign prostatic hyperplasia (BPH) and to study associations between androgens and histologic score of prostate tissue in PCa. Two hundred ten men older than 45 years selected from 2906 participants of a population screening for PCa were studied: 70 with PCa, 70 with BPH and 70 controls (CG), matched by body mass index and age. Insulin, IGF-1, PSA, |

| | |
|---|---|
| | leptin, total, free (fT) and bioavailable testosterone (bT) and estradiol were measured. Each group was subdivided into two subgroups considering the presence of metabolic syndrome (MS); androgens and leptin levels were analyzed in the subgroups. Prostate cancer and BPH patients presented higher total, fT and bT levels than CG. IGF-1, insulin and HOMA index were higher in BPH than in the other two groups. PCa presented higher leptin [median (range) 6.5 (1.3-28.0) versus 4.8 (1.1-12.3) ng/ml; $p < 0.01$] and estradiol [median (range) 37.0 (20-90) versus 29.0 (20-118) pg/ml; $p = 0.025$] levels than CG. After dividing men considering the presence of MS, leptin was higher and total testosterone was lower in MS patients in all the groups. It was observed a coexistence of an altered hormone profile with increased sex hormones and leptin in PCa patients, in accordance with the new perspective of PCa pathogenesis. |
| **Question** | Complex relationship between [........], insulin resistance and leptin in men with and without prostatic disease. |
| **Options** | benign prostatic hyperplasia / estradiol / associations / Insulin / PSA / men / leptin / testosterone / patients / population / prostate cancer / tissue / body mass index / PCa / syndrome / androgens / hormone / screening / insulin-resistance / IGF-1 / sex hormones / prostate / |
| **Answer** | sex hormones |
| **Response** | |
| **CS undergrad 1** | sex hormones (o) |
| **CS undergrad 2** | sex hormones (o) |
| **Bio. Grad 1** | hormone (x) |
| **Bio. Grad 2** | testosterone (x) |
| **Expert 1** | testosterone (x) |
| **Expert 2** | testosterone (x) |
| **ASR model** | sex hormones (o) |

Question 41.

| | |
|---|---|
| **Source dataset** | **BMKC_LS** |
| **Context** | Kaposi's sarcoma (KS), a highly angiogenic and invasive tumor often involving different organ sites, including the oral cavity, is caused by infection with Kaposi's sarcoma-associated herpesvirus (KSHV). Diverse cell markers have been identified on KS tumor cells, but their origin remains an enigma. We previously showed that KSHV could efficiently infect, transform, and reprogram rat primary mesenchymal stem cells (MSCs) into KS-like tumor cells. In this study, we showed that human primary |

| | |
|---|---|
| | MSCs derived from diverse organs, including bone marrow (MSCbm), adipose tissue (MSCa), dental pulp, gingiva tissue (GMSC), and exfoliated deciduous teeth, were permissive to KSHV infection. We successfully established long-term cultures of KSHV-infected MSCa, MSCbm, and GMSC (LTC-KMSCs). While LTC-KMSCs had lower proliferation rates than the uninfected cells, they expressed mixtures of KS markers and displayed differential angiogenic, invasive, and transforming phenotypes. Genetic analysis identified KSHV-derived microRNAs that mediated KSHV-induced angiogenic activity by activating the AKT pathway. These results indicated that human MSCs could be the KSHV target cells in vivo and established valid models for delineating the mechanism of KSHV infection, replication, and malignant transformation in biologically relevant cell types. Kaposi's sarcoma is the most common cancer in AIDS patients. While KSHV infection is required for the development of Kaposi's sarcoma, the origin of KSHV target cells remains unclear. We show that KSHV can efficiently infect human primary mesenchymal stem cells of diverse origins and reprogram them to acquire various degrees of Kaposi's sarcoma-like cell makers and angiogenic, invasive, and transforming phenotypes. |
| **Question** | These results indicate that [........] mesenchymal stem cells might be the KSHV target cells and establish models for delineating the mechanism of KSHV-induced malignant transformation. |
| **Options** | cells / cultures / adipose tissue / dental pulp / tumor / gingiva / mesenchymal stem cells / microRNAs / patients / tissue / phenotypes / KSHV / deciduous teeth / MSCs / human / AIDS / bone marrow / AKT / infection / rat / Kaposi's sarcoma / oral cavity / |
| **Answer** | human |
| **Response** | |
| **CS undergrad 1** | rat (x) |
| **CS undergrad 2** | KSHV (x) |
| **Bio. Grad 1** | human (o) |
| **Bio. Grad 2** | human (o) |
| **Expert 1** | human (o) |
| **Expert 2** | Kaposi's sarcoma (x) |
| **ASR model** | human (o) |

Question 42.

| | |
|---|---|
| **Source dataset** | **BMKC_T** |
| **Context** | Triple-negative breast cancer (TNBC) is a highly aggressive and |

| | |
|---|---|
| | recurrent type of breast carcinoma that is associated with poor patient prognosis. Because of the limited efficacy of current treatments, new therapeutic strategies need to be developed. The CXCR4-CXCL12 chemokine signaling axis guides cell migration in physiological and pathological processes, including breast cancer metastasis. Although targeted therapies to inhibit the CXCR4-CXCL12 axis are under clinical experimentation, still no effective therapeutic approaches have been established to block CXCR4 in TNBC. To unravel the role of the CXCR4-CXCL12 axis in the formation of TNBC early metastases, we used the zebrafish xenograft model. Importantly, we demonstrate that cross-communication between the zebrafish and human ligands and receptors takes place and human tumor cells expressing CXCR4 initiate early metastatic events by sensing zebrafish cognate ligands at the metastatic site. Taking advantage of the conserved intercommunication between human tumor cells and the zebrafish host, we blocked TNBC early metastatic events by chemical and genetic inhibition of CXCR4 signaling. We used IT1t, a potent CXCR4 antagonist, and show for the first time its promising anti-tumor effects. In conclusion, we confirm the validity of the zebrafish as a xenotransplantation model and propose a pharmacological approach to target CXCR4 in TNBC. |
| **Question** | Inhibition of signaling between human CXCR4 and zebrafish ligands by the small molecule [........] impairs the formation of triple-negative breast cancer early metastases in a zebrafish xenograft model. |
| **Options** | xenotransplantation / breast carcinoma / role / cells / CXCR4 / Triple-negative breast cancer / tumor / IT1t / communication / therapeutic / inhibition / metastases / patient / CXCL12 / prognosis / time / xenograft / human / cell migration / pathological processes / ligands / CXCL12 chemokine / zebrafish / |
| **Answer** | IT1t |
| **Response** | |
| **CS undergrad 1** | breast carcinoma (x) |
| **CS undergrad 2** | CXCL12 (x) |
| **Bio. Grad 1** | CXCL12 (x) |
| **Bio. Grad 2** | IT1t (o) |
| **Expert 1** | therapeutic (x) |
| **Expert 2** | IT1t (o) |
| **ASR model** | CXCR4 (x) |

Question 43.

| | |
|---|---|
| **Source dataset** | **BMKC_LS** |
| **Context** | Tumor-associated macrophages (TAMs), the most abundant infiltrating immune cells in tumor microenvironment, have distinct functions in hepatocellular carcinoma (HCC) progression. $CD68^+$ TAMs represent multiple polarized immune cells mainly containing $CD86^+$ antitumoral M1 macrophages and $CD206^+$ protumoral M2 macrophages. TAMs expression and density were assessed by immunohistochemical staining of CD68, CD86, and CD206 in tissue microarrays from 253 HCC patients. Clinicopathologic features and prognostic value of these markers were evaluated. We found that $CD68^+$ TAMs were not associated with clinicopathologic characteristics and prognosis in HCC. Low presence of $CD86^+$ TAMs and high presence of $CD206^+$ TAMs were markedly correlated with aggressive tumor phenotypes, such as multiple tumor number and advanced tumor-node-metastasis (TNM) stage; and were associated with poor overall survival (OS) ($p = 0.027$ and $p = 0.024$, respectively) and increased time to recurrence (TTR) ($p = 0.037$ and $p = 0.031$, respectively). In addition, combined analysis of CD86 and CD206 provided a better indicator for OS ($p = 0.011$) and TTR ($p = 0.024$) in HCC than individual analysis of CD86 and CD206. Moreover, $CD86^+/CD206^+$ TAMs predictive model also had significant prognosis value in α-fetoprotein (AFP)-negative patients (OS: $p = 0.002$, TTR: $p = 0.005$). |
| **Question** | Thus, these results suggest that combined analysis of immune biomarkers [........] and CD206 could be a promising HCC prognostic biomarker. |
| **Options** | HCC / staining / cells / Tumor / recurrence / tumor microenvironment / metastasis / patients / tissue / survival / TAMs / biomarkers / prognosis / phenotypes / time / overall / TTR / CD86 / hepatocellular carcinoma / CD206 / macrophages / CD68 / AFP / TNM / |
| **Answer** | CD86 |
| **Response** | |
| **CS undergrad 1** | macrophages (x) |
| **CS undergrad 2** | TAMs (x) |
| **Bio. Grad 1** | CD86 (o) |
| **Bio. Grad 2** | CD86 (o) |
| **Expert 1** | CD86 (o) |
| **Expert 2** | CD86 (o) |
| **ASR model** | Cells (x) |

Question 44.

| | |
|---|---|
| **Source dataset** | **BMKC_T** |
| **Context** | The dire effects of cancer-induced cachexia undermine treatment and contribute to decreased survival rates. Therapeutic options for this syndrome are limited, and therefore efforts to identify signs of precachexia in cancer patients are necessary for early intervention. The applications of molecular and functional imaging that would enable a whole-body "holistic" approach to this problem may lead to new insights and advances for diagnosis and treatment of this syndrome. Here we have developed a myoblast optical reporter system with the purpose of identifying early cachectic events. We generated a myoblast cell line expressing a dual tdTomato:GFP construct that was grafted onto the muscle of mice-bearing human pancreatic cancer xenografts to provide noninvasive live imaging of events associated with cancer-induced cachexia (i.e., weight loss). Real-time optical imaging detected a strong tdTomato fluorescent signal from skeletal muscle grafts in mice with weight losses of only 1.2% to 2.7% and tumor burdens of only approximately 79 to 170 mm(3). Weight loss in cachectic animals was also associated with a depletion of lipid, cholesterol, valine, and alanine levels, which may provide informative biomarkers of cachexia. Taken together, our findings demonstrate the utility of a reporter system that is capable of tracking tumor-induced weight loss, an early marker of cachexia. Future studies incorporating resected tissue from human pancreatic ductal adenocarcinoma into a reporter-carrying mouse may be able to provide a risk assessment of cachexia, with possible implications for therapeutic development. Cancer Res; 76(6); 1441-50. ©2015 AACR. |
| **Question** | Detection of Pancreatic [........]-Induced Cachexia Using a Fluorescent Myoblast Reporter System and Analysis of Metabolite Abundance. |
| **Options** | carrying / tumor burdens / skeletal muscle / adenocarcinoma / Future / tumor / Therapeutic / optical imaging / grafts / patients / mice / tissue / weight loss / biomarkers / syndrome / survival rates / time / xenografts / diagnosis / alanine / risk assessment / human / lead / cachexia / cholesterol / cell line / muscle / animals / early intervention / valine / myoblast / |
| **Answer** | tumor |
| **Response** | |
| **CS undergrad 1** | adenocarcinoma (x) |
| **CS undergrad 2** | adenocarcinoma (x) |

| | |
|---|---|
| **Bio. Grad 1** | tumor (o) |
| **Bio. Grad 2** | tumor (o) |
| **Expert 1** | tumor (o) |
| **Expert 2** | adenocarcinoma (x) |
| **ASR model** | tumor (o) |

Question 45.

| | |
|---|---|
| **Source dataset** | **BMKC_LS** |
| **Context** | In successful cancer immunotherapy, T cell responses appear to be directed toward neoantigens created by somatic mutations; however, direct evidence that neoantigen-specific T cells cause regression of established cancer is lacking. Here, we generated T cells expressing a mutation-specific transgenic T cell receptor (TCR) to target different immunogenic mutations in cyclin-dependent kinase 4 (CDK4) that naturally occur in human melanoma. Two mutant CDK4 isoforms (R24C, R24L) similarly stimulated T cell responses in vitro and were analyzed as therapeutic targets for TCR gene therapy. In a syngeneic HLA-A2-transgenic mouse model of large established tumors, we found that both mutations differed dramatically as targets for TCR-modified T cells in vivo. While T cells expanded efficiently and produced IFN-γ in response to R24L, R24C failed to induce an effective antitumor response. Such differences in neoantigen quality might explain why cancer immunotherapy induces tumor regression in some individuals, while others do not respond, despite similar mutational load. We confirmed the validity of the in vivo model by showing that the melan-A-specific (MART-1-specific) TCR DMF5 induces rejection of tumors expressing analog, but not native, MART-1 epitopes. |
| **Question** | The described model allows identification of those neoantigens in human [........] that serve as suitable T cell targets and may help to predict clinical efficacy. |
| **Options** | TCR gene / IFN-γ / tumors / melan-A / immunotherapy / therapeutic / identification / R24L / regression / cyclin-dependent kinase 4 / in vitro / isoforms / human / CDK4 / transgenic mouse / MART-1 / mutation / T cell receptor / epitopes / T cells / HLA-A2 / clinical efficacy / rejection / |
| **Answer** | cancer |
| **Response** | |
| **CS undergrad 1** | melan-A (x) |
| **CS undergrad 2** | CDK4 (x) |
| **Bio. Grad 1** | T cells (x) |

| | |
|---|---|
| **Bio. Grad 2** | immunotherapy (x) |
| **Expert 1** | tumors (x) |
| **Expert 2** | transgenic mouse (x) |
| **ASR model** | Cancer (o) |

Question 46.

| | |
|---|---|
| **Source dataset** | **BMKC_T** |
| **Context** | Transplantation of pluripotent stem cells and their differentiated progeny has the potential to preserve or regenerate functional pathways and improve function after central nervous system injury. However, their utility has been hampered by poor survival and the potential to form tumors. Peptide-modified biomaterials influence cell adhesion, survival and differentiation in vitro, but their effectiveness in vivo remains uncertain. We synthesized a peptide-modified, minimally invasive, injectable hydrogel comprised of hyaluronan and methylcellulose to enhance the survival and differentiation of human induced pluripotent stem cell-derived oligodendrocyte progenitor cells. Cells were transplanted subacutely after a moderate clip compression rat spinal cord injury. The hydrogel, modified with the RGD peptide and platelet-derived growth factor (PDGF-A), promoted early survival and integration of grafted cells. However, prolific teratoma formation was evident when cells were transplanted in media at longer survival times, indicating that either this cell line or the way in which it was cultured is unsuitable for human use. Interestingly, teratoma formation was attenuated when cells were transplanted in the hydrogel, where most cells differentiated to a glial phenotype. Thus, this hydrogel promoted cell survival and integration, and attenuated teratoma formation by promoting cell differentiation. |
| **Question** | Injectable [........] promotes early survival of induced pluripotent stem cell-derived oligodendrocytes and attenuates longterm teratoma formation in a spinal cord injury model. |
| **Options** | Transplantation / cells / PDGF-A / cell differentiation / pluripotent stem cells / tumors / injectable / cell survival / spinal cord injury / central nervous system / progenitor cells / survival / platelet-derived growth factor / phenotype / in vitro / cell adhesion / human / biomaterials / methylcellulose / oligodendrocyte / hyaluronan / hydrogel / rat / clip / cell line / teratoma / RGD peptide / |
| **Answer** | hydrogel |
| **Response** | |
| **CS undergrad 1** | hydrogel (o) |

| | |
|---|---|
| **CS undergrad 2** | hydrogel (o) |
| **Bio. Grad 1** | pluripotent stem cells (x) |
| **Bio. Grad 2** | hydrogel (o) |
| **Expert 1** | hydrogel (o) |
| **Expert 2** | hydrogel (o) |
| **ASR model** | hydrogel (o) |

Question 47.

| | |
|---|---|
| **Source dataset** | **BMKC_LS** |
| **Context** | Long noncoding RNAs (lncRNAs), which are epigenetic regulators, are involved in human malignancies. Little is known, however, about the molecular mechanisms for lncRNA regulation of genes induced by cigarette smoke. We recently found that, in human bronchial epithelial (HBE) cells, the lncRNA, Hox transcript antisense intergenic RNA (HOTAIR), is associated with changes in the cell cycle caused by cigarette smoke extract (CSE). In the present study, we report that increased expression of HOTAIR and enhancer of zeste homolog 2 (EZH2), and tri-methylation of Lys 27 of histone H3 (H3K27me3), affect cell cycle progression during CSE-induced transformation of HBE cells. Inhibition of HOTAIR and EZH2 by siRNAs attenuated CSE-induced decreases of p21 levels. Further, ChIP assays verified that HOTAIR and EZH2 were needed to maintain the interaction of H3K27me3 with the promoter regions of p21; combined use of a HOTAIR plasmid and EZH2 siRNA supported this observation. Thus, HOTAIR epigenetic silencing of p21 via EZH2-mediated H3K27 trimethylation contributes to changes in the cell cycle induced by CSE. |
| **Question** | These observations provide further understanding of the [........] of CSE-induced lung carcinogenesis and identify new therapeutic targets. |
| **Options** | affect / Long noncoding RNAs / cells / histone H3 / CSE / epigenetic / observations / cigarette / cell cycle / report / therapeutic / Inhibition / regulation / EZH2 / p21 / human / smoke / carcinogenesis / genes / methylation / siRNAs / understanding / lung / HOTAIR / plasmid / HBE / RNA / promoter regions / |
| **Answer** | regulation |
| **Response** | |
| **CS undergrad 1** | cell cycle (x) |
| **CS undergrad 2** | p21 (x) |

| | |
|---|---|
| **Bio. Grad 1** | affect (x) |
| **Bio. Grad 2** | cell cycle (x) |
| **Expert 1** | regulation (o) |
| **Expert 2** | epigenetic (x) |
| **ASR model** | regulation (o) |

Question 48.

| | |
|---|---|
| **Source dataset** | **BMKC_T** |
| **Context** | Colchicine is a tubulin-binding natural product isolated from Colchicum autumnale. Here we report the in vitro anticancer activity of C-ring modified semi-synthetic derivative of colchicine; N-[(7S)-1,2,3-trimethoxy-9-oxo-10-(4-phenyl-piperidin-1-yl)-5,6,7,9 tetrahydrobenzo[a]heptalen-7-yl]acetamide (4h) on colon cancer HCT-116 cell line. The compound 4h was screened for anti-proliferative activity against different human cancer cell lines and was found to exhibit higher cytotoxicity against colon cancer cell lines HCT-116 and Colo-205 with IC50 of 1 and 0.8 μM respectively. Cytotoxicity of the compound to the normal fR2 breast epithelial cells and normal HEK293 human embryonic kidney cells was evaluated in concentration and time-dependent manner to estimate its selectivity for cancer cells which showed much better selectivity than that of colchicine. Compound 4h induced cell death in HCT-116 cells by activating apoptosis and autophagy pathways. Autophagy inhibitor 3-MA blocked the production of LC3-II and reduced the cytotoxicity in response to 4h, but did not affect apoptosis, suggesting thereby that these two were independent events. Reactive oxygen species scavenger ascorbic acid pretreatment not only decreased the reactive oxygen species level but also reversed 4h induced cytotoxicity. Treatment with compound 4h depolymerized microtubules and the majority of cells arrested at the G2/M transition. Together, these data suggest that 4h has better selectivity and is a microtubule depolymerizer, which activates dual cell-death machineries, and thus, it could be a potential novel therapeutic agent in cancer therapy. Copyright © 2016 John Wiley & Sons, Ltd. |
| **Question** | A novel microtubule depolymerizing colchicine analogue triggers apoptosis and autophagy in HCT-116 colon [........] cells. |
| **Options** | affect / HCT-116 cell / ascorbic acid / Copyright / cells / concentration / autophagy / IC50 / breast / kidney / report / cancer / heptalen / Treatment / microtubules / Colo-205 / inhibitor 3 / cell-death / tubulin / time / in vitro / phenyl / Reactive oxygen species / human / Colchicine / Colchicum autumnale / Sons / production / cell lines / colon cancer / LC3 / |

| | |
|---|---|
| | HCT-116 / acetamide / apoptosis / |
| **Answer** | cancer |
| **Response** | |
| **CS undergrad 1** | kidney (x) |
| **CS undergrad 2** | cancer (o) |
| **Bio. Grad 1** | cancer (o) |
| **Bio. Grad 2** | cancer (o) |
| **Expert 1** | cancer (o) |
| **Expert 2** | cancer (o) |
| **ASR model** | cancer (o) |

Question 49.

| | |
|---|---|
| **Source dataset** | **BMKC_LS** |
| **Context** | : The treatment outcomes of myelodysplastic syndrome (MDS) and transformed acute myelogenous leukemia (tAML) remain very unsatisfactory. We designed a combination of human leukocyte antigen (HLA)-mismatched hematopoietic stem cell microtransplantation (MST) with chemotherapy for patients with MDS and tAML and evaluated its effects and toxicity. Patients were between 13 and 79 years old. Patients with MDS (n = 21) were given HLA-mismatched MST combined with decitabine and cytarabine; patients with tAML (n = 22) were given HLA-mismatched MST combined with decitabine and cytarabine, and also mitoxantrone. Patients in complete remission (CR) also received MST plus decitabine and medium-dose cytarabine chemotherapy without graft-versus-host disease (GVHD) prophylaxis. The overall response rate of the patients with MDS was significantly higher than that of those with tAML (81% vs. 50%; p = .03). The CR rates were 52.4% and 36.4% in the two groups, respectively. There was no difference in the cytogenetic CR rate between the MDS and tAML groups (85.7% vs. 70%, respectively; p = .7). The 24-month overall survival of the patients with MDS was significantly higher than that of the patients with tAML (84.7% and 34.1%, respectively; p = .003). The median recovery times of neutrophils and platelets were, respectively, 14 and 17 days in the patients with MDS, and 16 and 19 days in those with tAML. The treatment-related mortality rates were 4.8% and 18.2%, respectively, in the MDS and tAML groups (p = .34). No GVHD was observed in any patient. Microtransplantation combined with decitabine and chemotherapy may provide a novel, effective, and safe treatment for high-risk MDS and tAML. Microtransplantation (MST) refers to regular chemotherapy |

| | |
|---|---|
| | combined with granulocyte colony-stimulating factor-mobilized peripheral blood stem cell infusion of human leukocyte antigen-mismatched donor cells without using immunosuppressive agents. It aims to support hematopoietic recovery and perform graft-versus-leukemia (GVL) effects but differs from traditional allogeneic stem cell transplantation because the rate of donor cell chimerism is low and there is and no graft-versus-host disease (GVHD) risk. Thus, a trial was designed to evaluate the safety and efficacy of MST in patients with myelodysplastic syndrome and those with transformed acute myelogenous leukemia. |
| **Question** | Higher complete remission and [........] complete response rates were observed, and the treatment improved disease progress-free survival, sped hematopoietic recovery, and avoided GVHD. |
| **Options** | acute myelogenous leukemia / cell / chemotherapy / MST / hematopoietic stem cell / neutrophils / decitabine / safety / risk / cytogenetic / mortality rates / immunosuppressive agents / cytarabine / treatment / disease / stem cell transplantation / Patients / graft / survival / leukocyte antigen / times / overall / leukemia / human / platelets / chimerism / mitoxantrone / graft-versus-host disease / granulocyte colony-stimulating factor / donor / treatment outcomes / myelodysplastic syndrome /<br>c |
| **Answer** | cytogenetic |
| **Response** | |
| **CS undergrad 1** | MST (x) |
| **CS undergrad 2** | treatment (x) |
| **Bio. Grad 1** | overall (x) |
| **Bio. Grad 2** | overall (x) |
| **Expert 1** | overall (x) |
| **Expert 2** | chemotherapy (x) |
| **ASR model** | Cytogenetic (o) |

Question 50.

| | |
|---|---|
| **Source dataset** | **BMKC_T** |
| **Context** | The aim of the present study was to investigate the effects of dextran sulphate (DS) on HIF-1α and integrin β1 (ITGβ1) expression in human gastric cancer cells, the correlation between HIF-1α and ITGβ1 expression and the influence of DS on the peritoneal metastasis of human gastric cancer cells. In in vitro experiments, BGC-823 cells in the experimental and control groups were administered DS and PBS, respectively, and exposed |

| | |
|---|---|
| | to hypoxic conditions for different periods. Immunocytochemistry, western blot and RT-PCR analyses were used to evaluate HIF-1α and ITGβ1 expression levels. In in vivo experiments, an animal model was established by injecting BGC-823 cells into nude mice. The experimental and control groups received DS and PBS injections, respectively. The mice were euthanized at different times, and the number of tumor nodules in the celiac implantation was recorded. Immunohistochemistry, RT-PCR and western blot analyses were used to detect HIF-1α and ITGβ1 expression in the tumor nodules of the greater omentum. The in vitro and in vivo results revealed that HIF-1α and ITGβ1 expression levels in the experimental group were significantly lower than those in the control group ($P<0.05$), and the expression levels of these factors were positively correlated with each other. The number of tumor nodules in the in vivo experiments was notably less in the experimental group than that noted in the control group ($P<0.01$). In conclusion, DS may act through inhibition of HIF-1α expression, which decreased ITGβ1 expression, consequently reducing tumor metastasis. |
| **Question** | Inhibition of peritoneal metastasis of human gastric cancer [........] by dextran sulphate through the reduction in HIF-1α and ITGβ1 expression. |
| **Options** | nude mice / gastric cancer / western blot / cells / BGC-823 / sulphate / control groups / tumor / injections / inhibition / metastasis / mice / times / in vitro / dextran / PCR / human / animal model / PBS / omentum / Immunocytochemistry / |
| **Answer** | cells |
| **Response** | |
| **CS undergrad 1** | control groups (x) |
| **CS undergrad 2** | cells (o) |
| **Bio. Grad 1** | cells (o) |
| **Bio. Grad 2** | cells (o) |
| **Expert 1** | cells (o) |
| **Expert 2** | cells (o) |
| **ASR model** | cells (o) |
