# Supplementary material for: A Pilot Study of Biomedical Text Comprehension using an Attention-Based Deep Neural Reader: Design and Experimental Analysis
Source: JMIR Med Inform. 2018 Jan 5;6(1):e2. doi: 10.2196/medinform.8751 (PMC5783222; doi:10.2196/medinform.8751)
Supplement: Multimedia Appendix 2 [file medinform_v6i1e2_app2.pdf]

Multimedia Appendix 1

Human evaluation of the CNN and CBT dataset using crowdsourcing

In this section, we describe in detail the human comprehension performance experiments on the CNN and CBT datasets using crowdsourcing. We allocate a human evaluator to evaluate questions from both datasets. For our evaluation, we randomly extracted 50 questions from each of the two datasets. For the crowdsourcing platform, we used CrowdFlower (https://www.crowdflower.com/).

We assigned 6 to 12 random questions to a human evaluator from our question pool. The number of questions to solve is decided by an evaluator. We validated human evaluators by training and testing them with a sample subset of questions. The validation process is meaningful in that participants can be well acquainted with our actual task and we can filter the unsuitable evaluators at the same time.

All evaluators that did not exceed 50% in accuracy on our sample set could not participate in our evaluation process. We did not limit the geo-location of the evaluators. The verified evaluators are paid $0.15 per question. To calibrate our test results, we assign ten human evaluators to a single question. The evaluation results from a question are then averaged. For example, if 7 out of 10 evaluators correctly answered a question, the accuracy is 0.7. We simply add up the averaged result from each question, which makes up to total of 50 questions for each dataset.
